# Characterization of the Phospholipid Platelet-Activating Factor As a Mediator of Inflammation in Chickens

**DOI:** 10.3389/fvets.2017.00226

**Published:** 2017-12-18

**Authors:** Damien Garrido, Nathalie K. Chanteloup, Angélina Trotereau, Adrien Lion, Geoffrey Bailleul, Evelyne Esnault, Sascha Trapp, Pascale Quéré, Catherine Schouler, Rodrigo Guabiraba

**Affiliations:** ^1^ISP, INRA, Université François Rabelais de Tours, Nouzilly, France

**Keywords:** avian pathogenic *Escherichia coli*, chicken, colibacillosis, endothelial cells, inflammation, macrophages, platelet-activating factor, platelet-activating factor receptor

## Abstract

Lipid mediators are known to play important roles in the onset and resolution phases of the inflammatory response in mammals. The phospholipid platelet-activating factor (PAF) is a pro-inflammatory lipid mediator which participates in vascular- and innate immunity-associated processes by increasing vascular permeability, by facilitating leukocyte adhesion to the endothelium, and by contributing to phagocyte activation. PAF exerts its function upon binding to its specific receptor, PAF receptor (PAFR), which is abundantly expressed in leukocytes and endothelial cells (ECs). In chickens, lipid mediators and their functions are still poorly characterized, and the role of PAF as an inflammatory mediator has not yet been investigated. In the present study we demonstrate that primary chicken macrophages express PAFR and lysophosphatidylcholine acyltransferase 2 (LPCAT2), the latter being essential to PAF biosynthesis during inflammation. Also, exogenous PAF treatment induces intracellular calcium increase, reactive oxygen species release, and increased phagocytosis by primary chicken macrophages in a PAFR-dependent manner. We also show that PAF contributes to the *Escherichia coli* lipopolysaccharide (LPS)-induced pro-inflammatory response and boosts the macrophage response to *E. coli* LPS *via* phosphatidylinositol 3-kinase/Akt- and calmodulin kinase II-mediated intracellular signaling pathways. Exogenous PAF treatment also increases avian pathogenic *E. coli* intracellular killing by chicken macrophages, and PAFR and LPCAT2 are upregulated in chicken lungs and liver during experimental pulmonary colibacillosis. Finally, exogenous PAF treatment increases cell permeability and upregulates the expression of genes coding for proteins involved in leukocyte adhesion to the endothelium in primary chicken endothelial cells (chAEC). In addition to these vascular phenomena, PAF boosts the chAEC inflammatory response to bacteria-associated molecular patterns in a PAFR-dependent manner. In conclusion, we identified PAF as an inflammation amplifier in chicken macrophages and ECs, which suggests that PAF could play important roles in the endothelium-innate immunity interface in birds during major bacterial infectious diseases such as colibacillosis.

## Introduction

Inflammation is a complex and a tightly coordinated biological process driven by a wide array of cells and chemical mediators (1). Lipid mediators (e.g., eicosanoids and phospholipids) are molecules well known to take part in the onset of the inflammatory response in different vertebrate organisms (2, 3). These molecules are rapidly biosynthesized by myeloid and epithelial cells within seconds to minutes of acute stimulation of different origins (1, 4). Among these molecules, the phospholipid platelet-activating factor (PAF) potent pro-inflammatory lipid mediator. PAF activates a G protein-coupled receptor named PAF receptor (PAFR) (5–7), which results in pleiotropic and potent biological effects including platelet activation, airway constriction, hypotension, and increased vascular permeability *via* signaling pathways such as phosphatidylinositol 3-kinase (PI3K), PKC, phospholipase A2 (PLA_2_), and PLC (5, 8–10). PAF is synthesized *via* two distinct pathways, the *de novo* and remodeling pathways (2, 5, 11), the latter of which is regulated by inflammatory extracellular signals and plays a key role in PAF-induced physiopathology (2, 5, 11, 12). PAF biosynthesis occurs through lyso-PAF formation *via* PLA_2_ catalytic activity. In a subsequent step, lysophosphatidylcholine acyltransferase (LPCAT, a member of the lysophospholipid acyltransferase family) generates PAF from lyso-PAF. LPCAT2 is known to biosynthesize PAF upon pro-inflammatory stimuli and may localize to the endoplasmic reticulum (ER) and the Golgi (13, 14). Moreover, PAF levels in the extracellular milieu can be controlled through rapid degradation by PAF-acetylhydrolase (PAF-AH) (15). All these physiological or pathological processes have been well characterized in mammals, more notably in rodents and humans.

Platelet-activating factor synthesis upon inflammatory stimuli such as bacterial lipopolysaccharide (LPS) has been reported in murine peritoneal cells and macrophages (16, 17) and in human eosinophils and neutrophils (18, 19). PAF has been shown to be particularly active in mammalian macrophages during inflammation. It can reprogram macrophages such that there is an enhanced cytokine response to subsequent endotoxin stimulation (20). In addition, PAF acts as an autocrine mediator of macrophage activation upon LPS challenge *via* tumor necrosis factor alpha (TNF-α) expression (21). Systemic administration of PAF mimics a septic shock condition characterized by vasodilation with diminished peripheral vascular resistance and tissue oxygen utilization (22, 23). Moreover, TNF-α and PAF act synergistically to increase neutrophil–endothelium adhesion by stimulating endothelial expression of cell adhesion molecules: intercellular adhesion molecule 1 and E-selectin (24).

Phylogenetically, the regulatory role of PAF is presumed to have appeared in protozoans, having been maintained during evolution (25). In addition to mammals, PAF biosynthesis has been found in marine invertebrates and lower vertebrates (e.g., birds and reptiles). In chickens, PAF production has been observed in the chick’s retina (26). Also, PAF has been shown to activate chicken thrombocytes in a Ca^2+^-dependent manner (27). Enzymes involved in the metabolism and catabolism of PAF in chickens were poorly studied, although PAF-AH was reported to be absent in chicken serum (28). Nevertheless, catalytic residues in PAF-AH sequence are conserved in chickens (29). Overall, very few studies specifically addressed the role of inflammatory lipid mediators in chickens, although some data are available for eicosanoids (30–32). However, to date, no study has addressed the putative regulatory roles for PAF in chickens during inflammation or infection.

Poultry flocks are often impacted by several infectious diseases, and avian colibacillosis is the most prevalent bacterial infection, representing important economic losses to the poultry industry worldwide (33). Colibacillosis is caused by avian pathogenic *Escherichia coli* (APEC) strains, commonly called APEC. Antibiotherapy is leading to antimicrobial resistance, and commercially available vaccines confer protection to a limited set of APEC strains. Pulmonary colibacillosis is the most common form of disease, characterized by acute inflammatory response with massive influx of heterophils and macrophages. Although colibacillosis pathogenesis needs to be better characterized, it is believed that macrophages are crucially involved in bacterial killing and amplification of inflammation in APEC infected chicken lungs (33–35).

In line with that, PAF has been implicated in several animal models of lung injury, and treatment with PAF receptor antagonists attenuates endotoxin-induced lung injury (36). During pulmonary Gram-negative bacteria infection, PAF production is important for bacteria clearance (37). However, during experimental influenza A virus infection in mice, PAF plays a deleterious role by amplifying inflammation and tissue damage (38).

Despite a growing interest in characterizing early mediators of inflammation in chickens, and more notably their crosstalk with innate immunity sentinel cells such as macrophages, no previous works have addressed the mechanisms by which lipid mediators may contribute to inflammation or bacterial clearance. In the present work, we aimed to characterize the PAF/PAFR interactions and to shed light on their functions in chicken macrophages and endothelial cells (ECs), especially with respect to how PAF system molecules are modulated during pulmonary colibacillosis.

## Materials and Methods

### Reagents

Platelet-activating factor (synthetic PAF C16:0, β-acetyl-γ-*O*-hexadecyl-l-α-phosphatidylcholine hydrate, ≥98% HPLC purity tested), human PAF-AH, dimethyl sulfoxide (DMSO), LPS (from *E. coli* O55:B5), and 2′,7′-dichlorofluorescein diacetate (DCFDA, ≥95% HPLC purity tested) were purchased from Sigma-Aldrich, UK. PAFR antagonists PCA 4248 (C_19_H_23_NO_4_S, >95% HPLC purity tested) and WEB 2086 (Apafant, C_22_H_22_N_5_O_2_SCl, >99% HPLC purity tested), PI3K/Akt inhibitor wortmannin (WMN; SL-2052, C_23_H_24_O_8_), and calcium calmodulin kinase II (CaMK II) inhibitor KN62 (C_38_H_35_N_5_O_6_S_2_, >98% HPLC purity tested) were purchased from Tocris Biotechne, UK.

### Animals

White leghorn chickens from the GB1 Athens inbred line (B13/B13 histocompatible chickens) or from the PA12 outbred line were hatched and raised specific-pathogen-free (SPF) at INRA (Plate-Forme d’Infectiologie Expérimentale, PFIE, Nouzilly, France). Food and water were provided *ad libitum*. For APEC infection experiments, B13/B13 histocompatible chickens were housed in BSL2 poultry isolator units.

### Cell Culture

HD11, an avian myelocytomatosis virus (MC29)-transformed chicken macrophage-like cell line (39), was cultured in RPMI-1640 medium (Gibco, UK) supplemented with 10% heat-inactivated fetal calf serum (FCS, Gibco, UK), 10 mM HEPES, 2 mM glutamine, 100 U/ml penicillin, and 100 µg/ml streptomycin (all from Sigma-Aldrich, UK) and grown routinely in a 75-cm^2^ flask (Corning, NY, USA) at 40°C under 5% CO_2_.

Chicken bone marrow-derived macrophages (BMDM) were outgrown from bone marrow cells using recombinant chicken macrophage colony-stimulating factor 1 (CSF-1) (40) derived from COS-7 cells (fibroblast-like cell line derived from monkey kidney tissue, ATCC, USA) transfected with a pTArget vector (Promega, UK) expressing chicken CSF-1 (kindly provided by Prof. Pete Kaiser, The Roslin Institute, UK). Briefly, femurs and tibia of 3–4-week-old B13/B13 histocompatible or PA12 chickens were removed, cut, and the marrow flushed with sterile phosphate-buffered saline (PBS, Gibco, UK). Next, cells were resuspended in RPMI-1640, loaded onto an equal volume of Histopaque 1.077 gradient (Sigma-Aldrich, UK), and centrifuged at 250 *g* for 30 min without brake. Cells at the interface were collected, washed twice with PBS, and cultured at a final concentration of 1 × 10^6^ cells/ml in sterile 60 mm bacteriological plates (Corning, NY, USA) in prewarmed complete RPMI-1640 medium supplemented with COS-7 supernatant containing chicken CSF-1, 10% FCS, 10 mM HEPES, 2 mM glutamine, 100 U/ml penicillin, and 100 µg/ml streptomycin at 40°C under 5% CO_2_. Half of the medium was replaced with fresh, pre-warmed complete medium containing chicken CSF-1 at day 4. At day 7, adherent cells were washed and harvested in cold PBS containing 2 mM ethylenediaminetetraacetic acid (EDTA, Sigma-Aldrich, UK), centrifuged, and resuspended in complete RPMI-1640 medium. All adherent cells presented macrophage-like morphology as evaluated in glass slides prepared in a CytoSpin^®^ Cytocentrifuge (Shandon, USA). Also, 97% of these cells were KUL01+ cells (a chicken macrophage marker) as evaluated by Flow Cytometry using a mouse anti-chicken monoclonal antibody (1:200 dilution, Bio-Rad, UK) and a rat-anti mouse phycoerythrin-coupled secondary antibody (1:300 dilution, Biolegend, UK).

Peripheral blood mononuclear cells (PBMC) were isolated from B13/B13 histocompatible chickens as previously described (41). Briefly, peripheral blood collected in EDTA-coated tubes was mixed with 1% methylcellulose (Sigma-Aldrich, UK, 1:1 v/v) and centrifuged at 25 *g* for 15 min. The upper phase was then diluted with PBS to the original volume of blood/methylcellulose mixture, carefully layered onto a Histopaque 1.077 gradient and centrifuged at 250 *g* for 30 min without brake. The PBMC-containing layer was collected, washed, and resuspended in complete RPMI-1640 medium for further incubation at 40°C under 5% CO_2_. After 24 h, adherent cells presented monocyte/macrophage-like morphology as evaluated in glass slides prepared in a CytoSpin^®^ Cytocentrifuge (Shandon, USA).

Primary chicken aortic endothelial cells (chAEC) were prepared from specific-pathogen-free (SPF) 18-day-old PA12 chicken embryos as previously described (42). Primary chAEC were serially passaged (p2–p5) using 0.05% trypsin-EDTA (Gibco, UK) and kept in EC growth medium (EGM-2 BulletKit, LONZA) at 40°C under 5% CO_2_.

### General *In Vitro* Stimulation Protocol

Twelve-well tissue culture plates (Corning, NY, USA) were seeded at 7 × 10^5^ cells per well of HD-11 macrophage-like cells, BMDM, PBMC, or primary chAEC in complete medium and incubated at 40°C under 5% CO_2_ overnight prior to different stimulation protocols. LPS (10 ng/ml) and hPAF-AH (10 µg/ml) were previously diluted in RPMI-1640. PAF (0.1–10 µM), PCA 4248 (10 µM), WEB 2086 (10 µM), WMN (200 nM), and KN62 (1 µM) were previously diluted in DMSO. Dead bacteria [DB; APEC strain BEN2908 at 10 multiplicity of infection (MOI), 15 min incubation at 65°C for killing] were previously diluted in LB medium. Final concentration of DMSO (the vehicle used for the dilution of PAF, PAFR antagonists, and cell signaling inhibitors) in cell culture wells never exceeded 0.1%. Treatment with PAF receptor antagonists or intracellular signaling pathway inhibitors was performed 1 h prior to stimulation. Cell culture supernatants were recovered 6 h after stimulation (unless otherwise indicated) and stocked at −20 or −80°C for subsequent analyses specified below. Cells were washed in sterile PBS and lysed with RNA lysis buffer (Macherey-Nagel, Germany) containing 2-mercaptoethanol (Merck Millipore, Germany), snap frozen in liquid nitrogen, and stocked at −80°C until RNA extraction. For total protein quantification and Western blot (WB) analysis, cells were washed in PBS followed by cell lysis using RIPA buffer containing a cocktail of protease inhibitors, which also included phenylmethylsulfonyl fluoride and sodium orthovanadate (Santa Cruz Biotechnology, USA), and stocked at −20°C.

### Gene Expression Analysis

Total RNA was extracted from frozen cell lysates using the NucleoSpin RNA II kit (Macherey-Nagel, Germany) according to the manufacturer’s instructions. Tissue samples were first processed using Trizol (TRI Reagent, Sigma-Aldrich, UK), and RNA was further purified as for cell lysates. RNA quality and concentration were determined using a NanoDrop (Thermo Scientific, USA). Total RNA (up to 1 µg per reaction) was reverse transcribed with iScript cDNA synthesis kit (Bio-Rad, USA). Quantitative real-time PCR (qRT-PCR) was performed on a CFX96 machine (Bio-Rad, USA), the reaction mixture consisting of 1 µl of cDNA, 5 µl of iQ SYBR Green Supermix (Bio-Rad, USA), 0,25 µl of each primer pair (250 nM, Eurogentec, Belgium), and 3,5 µl of nuclease-free water (Sigma-Aldrich, UK) in a total reaction volume of 10 µl. qRT-PCR data were analyzed using the CFX Manager software 3.1 (Bio-Rad, USA). Amplicon size of qRT-PCR products was confirmed in a 2% agarose gel. Gene expression for each target gene was normalized to gene expression levels of chicken hypoxanthine-guanine phosphoribosyltransferase and β-2-microglobulin (β-2-M). Relative normalized expression was calculated using the 2^−ΔΔCt^ method, and data are represented as fold increase as compared to control group (unless otherwise indicated). qRT-PCR primers (Table 1) were designed and verified using primer BLAST.^1^

**Table 1:** Primer pairs used in the present study for quantitative real-time PCR analysis.

Target mRNA	Forward primer 5′–3′	Reverse primer 5′–3′
β-2-M (*B2M*)	CGTCCTCAACTGCTTCGCG	TCTCGTGCTCCACCTTGC
COX-2 (*PTGS2*)	CTGCTCCCTCCCATGTCAGA	CACGTGAAGAATTCCGGTGTT
CXCLi2 (*IL8L2*)	CTGCGGTGCCAGTGCATTAG	AGCACACCTCTCTTCCATCC
Hypoxanthine-guanine phosphoribosyltransferase (*HPRT*)	TGGTGGGGATGACCTCTCAA	GGCCGATATCCCACACTTCG
E-selectin (*SELE*)	AATGCAAAGCTGTGACCTGC	GCGTGGATTGTCCTGTCAGA
IL-1β (*IL-1B*)	AGGCTCAACATTGCGCTGTA	CTTGTAGCCCTTGATGCCCA
iNOS (*NOS2*)	CCACCAGGAGATGTTGAACTATGTC	CCAGATGTGTGTTTTCCATGCA
LPCAT2 (*LPCAT2*)	GTCCGACAAACCATGGCAAC	TCCAATTCGTCCCCCTTTGG
Platelet-activating factor receptor (*PTAFR*)	GGAGACCTTTCAGACCGTGG	TTTGCCCTCTCCAGACATCG
VCAM-1 (*VCAM1*)	AGCTTGATGTCAAAGTTCCTCCT	AGGTTCCATTGACTGCTGGT

### Intracellular Calcium

The Fluo-4 NW Calcium Assay kit (Molecular Probes, USA) was used to measure changes in HD11 cells’ intracellular calcium levels upon different stimuli. Briefly, HD11 cells at 3 × 10^4^ cells per well in a black 96-multiwell plate (Corning, NY, USA) were loaded with 100 µl of the dye loading solution containing Fluo-4 NW dye and probenecid, according to the manufacturer’s instructions. The 96-well plate was incubated at 40°C under 5% CO_2_ for 30 min in the dark, and the stimuli of interest were added to the cells at *T* = 0, and the plates were read immediately. The changes in Fluo-4 NW fluorescence were measured at an excitation wavelength of 494 nm and an emission wavelength of 516 nm in a GloMax^®^-Multi Detection System plate reader (Promega, UK). Calcium mobilization was recorded over time (213 s, with 3 s intervals).

### Apoptosis and Cell Viability

To evaluate the pro-apoptotic and cytotoxic effects of different stimuli, cells were cultured as previously mentioned. Apoptotic/late apoptotic cells were identified using annexin V and propidium iodide (PI) double-positive staining strategy. Briefly, following different stimuli, BMDM were washed with EDTA-containing-PBS, harvested, washed in cold PBS, and resuspended in an annexin-binding buffer (BD Biosciences). Subsequently, annexin V-fluorescein isothiocyanate (FITC) was added to the cells for 30 min. After a washing step, the cells were further incubated with PI (BD Biosciences) for 15 min. After two washing steps, stained cells (50,000 acquired events over total gated cells) were analyzed immediately using a BD FACSCalibur™ (BD Biosciences).

In other experiments, cell viability was determined using the colorimetric methylthiazoletetrazolium bromide (MTT) assay (Sigma-Aldrich, UK) that measures metabolic activity as a surrogate for cell viability/cytotoxicity. Briefly, following different stimuli, MTT was added to a final concentration of 5 μg/ml per well, and cells were incubated for 2 h at 40°C under 5% CO_2_. After complete solubilization of the dye using DMSO, plates were read at 550 nm in a Multiskan Ascent plate reader (Thermo Fisher Scientific, USA).

### Caspase 3/7 Activity

Caspase 3/7 activity was carried out using the Caspase-Glo 3/7 assay kit (Promega, UK) according to the manufacturer’s protocol. Briefly, BMDM at 3 × 10^4^ cells per well in a black 96-multiwell plate (Corning, NY, USA) were incubated with different stimuli for 6 h. Then, 100 µl of Caspase-Glo 3/7 reagent was added to the wells. Plates were gently shaken and then incubated in the dark at 20°C for 60 min before luciferase activity was recorded using a GloMax^®^-Multi Detection System plate reader (Promega, UK).

### Intracellular Reactive Oxygen Species (ROS) Analysis

The cell-penetrating dye 2′,7′-dichlorofluorescein diacetate (H_2_DCFDA) is oxidized by ROS to yield the fluorescent molecule 2′7′-dichlorofluorescein (DCFDA), which is retained intracellularly and visible at the 488 nm excitation laser line. To evaluate ROS production upon different stimuli, BMDM were cultured as previously described and incubated with 10 µM H_2_DCFDA (Sigma-Aldrich, UK) in serum-free medium for 20 min. Then medium was changed, and cells receiving different stimuli were kept at 40°C under 5% CO_2_ for 2 h. Cells were then collected and washed in ice-cold PBS prior to flow cytometry analysis (50,000 acquired events over total gated cells) using a BD FACSCalibur™ (BD Biosciences, UK).

### Flow Cytometry Analysis of PAFR

For the identification of PAFR expression in BMDM, cells were cultured as previously mentioned, exposed or not to different stimuli, and incubated for 2 h at 40°C under 5% CO_2_. Then, cells were harvested, washed in FACS buffer (2 mM EDTA and 2% FCS in PBS), centrifuged, resuspended in FACS buffer, and stained with a rabbit polyclonal antibody raised against amino acids 1–300 of PAFR of human origin (Santa Cruz Biotechnology, USA) at 1:200 dilution. After 45 min at 4°C, cells were washed, resuspended in FACS buffer, and stained with a goat anti-rabbit IgG-FITC (Santa Cruz Biotechnology, USA) at 1:500 dilution. After 40 min at 4°C, cells were washed, centrifuged, resuspended in FACS buffer, and analyzed by flow cytometry (100,000 acquired events over total gated cells) using a BD FACSCalibur™ (BD Biosciences, UK).

### Immunofluorescence

Bone marrow-derived macrophages or HD11 cells were grown at 2 × 10^5^ cells per well on sterile glass coverslips placed in 24-well plates containing complete medium and incubated at 40°C under 5% CO_2_ overnight. After adding the stimuli of interest for 2 h, cells were washed in PBS and fixed with 4% paraformaldehyde (Sigma-Aldrich, UK) for 20 min at room temperature, permeabilized with 0.5% Triton X-100 (Sigma-Aldrich, UK) for 5 min, and blocked with PBS, 0.1% Triton X-100, and 2% bovine serum albumin (Sigma-Aldrich, UK). Immunostaining was performed with a rabbit polyclonal antibody raised against amino acids 1–300 of PAFR of human origin or rabbit polyclonal IgG as an isotype control (Santa Cruz Biotechnology, USA) at 1:200 dilution. Secondary antibody goat anti-rabbit IgG-FITC (Santa Cruz Biotechnology, USA) was used at 1:500 dilution. Cell nuclei were counterstained with ProLong Gold Antifade Mountant with DAPI (Thermo Fisher, USA). Cells were observed under an Axiovert 200 M inverted epifluorescence microscope equipped with the Apotome imaging system (Zeiss, Germany). Images were captured with an Axiocam MRm camera and analyzed using the Axiovision software (Zeiss, Germany).

### Western Blot

Total protein from BMDM cell lysates was quantified using a Quick Start™ Bradford Protein Assay (Bio-Rad, USA). Thirty micrograms of protein from whole cell lysates were mixed with 2× electrophoresis sample buffer (Santa Cruz Biotechnology, USA). After boiling for 5 min, proteins were separated using 4–15% Mini-PROTEAN TGX™ Precast gels (Bio-Rad, USA) and transferred onto polyvinylidene difluoride (PVDF) membranes using a Trans-Blot^®^ Turbo™ Mini PVDF Transfer pack (Bio-Rad, USA) in a Trans-Blot^®^ Turbo™ Transfer system (Bio-Rad, USA). The membranes were blocked in a buffer containing 5% non-fat dry milk in PBS for 1 h. Primary antibody targeting PAFR was the same as utilized for immunofluorescence (IF) staining at 1:500 dilution. To detect Akt, a rabbit anti-Akt IgG polyclonal antibody (detecting endogenous levels of total Akt1, Akt2, and Akt3 proteins—Cell Signaling Technology, USA) was utilized at 1:500 dilution. To detect CaMK II, a rabbit anti-CaMK II IgG polyclonal antibody (epitope corresponding to amino acids 1–300 mapping at the N-terminus of CaMK IIα of human origin, Santa Cruz Biotechnology, USA) was utilized at 1:500. Mouse anti-glyceraldehyde-3-phosphate dehydrogenase IgG1 monoclonal antibody (GAPDH, Merck Millipore, Germany) was utilized as internal loading control at 1:1,000 dilution. After blocking, primary antibodies diluted in 5% non-fat dry milk in PBS were added to the membranes for incubation overnight at 4°C under agitation. Following reaction with the primary antibodies, membranes were thoroughly washed in wash buffer containing 0.1% Tween 20 (Sigma-Aldrich, UK) in PBS and labeled with HRP-conjugated secondary antibodies (goat anti-rabbit or rabbit anti-mouse, Life Technologies, USA, at 1:2,000 dilution) for 1 h. After washing, the antigens were visualized using a Clarity™ ECL Western Blotting Substrate (Bio-Rad, USA) in a chemiluminescence FUSION FX apparatus (Vilber Lourmat, France).

### Phagocytosis Assay

Bone marrow-derived macrophages were seeded at 2 × 10^5^ cells per well in 24-well tissue culture plates, treated with different stimuli and immediately incubated with 10 µg/ml of Zymosan A (*Saccharomyces cerevisiae*) BioParticles™ Alexa Fluor™ 488 conjugate (Thermo Fisher Scientific, USA) in complete medium at 4°C or 40°C under 5% CO_2_ for 1 h. After incubation, cells were harvested, washed twice with ice-cold EDTA-containing PBS, centrifuged, resuspended in PBS, and analyzed by flow cytometry (100,000 acquired events over total gated cells) using a BD FACSCalibur™ (BD Biosciences, UK). Data were expressed as the percentage difference (delta) between fluorescence positive cells and cells incubated at 40°C (active phagocytosis) and 4°C (limited phagocytosis).

### Nitrite Dosage

Nitrite (NaNO_2_) content was used as an index of nitric oxide (NO) production by chicken macrophages. Nitrite levels were determined in supernatants of BMDM, PBMC, or HD11 cell cultures by spectrophotometry using the Griess reagent system (Promega, UK). Briefly, 50 µl of the samples were added to 50 µl of freshly prepared Griess reagent in a 96-well plate. The absorbance was read at 550 nm in a Multiskan Ascent plate reader (Thermo Fisher Scientific, USA). The nitrite concentration was calculated using a sodium nitrite standard curve.

### Lipidomics Analysis

Prostaglandin E2 (PGE2) was quantified from BMDM supernatant using liquid chromatography–tandem mass spectrometry (LC-MS/MS). Briefly, 1 ml of BMDM cell culture supernatant was added to 300 µl of cold methanol and 5 µl of internal standards (deuterium labeled compounds). Samples were centrifuged at 900 *g* for 15 min at 4°C, supernatants were diluted in H_2_O to 2 ml and submitted to solid phase extraction using a HRX 96-well plate (50 mg/well, Macherey Nagel, Germany). LC-MS/MS analyses were performed on a Agilent 1290 Infinity HPLC system coupled to an ESI-triple quadruple G6460 mass spectrometer (Agilent Technologies) as previously described (43).

### *In Vitro* Infection Protocol

HD11 cells were seeded in 12-well plates at 5 × 10^5^ cells per well and incubated at 40°C under 5% CO_2_ overnight. APEC strains BEN2908 [O2:K1:H5, a nalidixic acid-resistant derivative of strain MT78 which was isolated from the trachea of a chicken with respiratory infection (44)] and BEN3421 (O2:K1:H4, a nalidixic acid- and tetracyclin-resistant strain isolated from a chicken presenting colibacillosis in 2009 in France) were utilized for infection. Cell monolayers were infected with mid-log-phase bacteria at an MOI of 10 and incubated for 1 h in cell culture medium without FCS. Cells were washed and remaining extracellular bacteria were killed by incubation in complete medium containing gentamicin (100 µg/ml) for 1 h 30 min. A group of cells were then washed and lysed with sterile water for 30 min at 4°C to evaluate the percentage of invasion through bacterial enumeration by viable counts on LB agar plates. Percentage of invasion (*T* = 0) was calculated as the number of intracellular bacteria divided by the number of bacteria in the inoculum. For the other groups, culture medium was changed, and gentamicin concentration was reduced to 10 µg/ml (a concentration known to kill BEN2908 and BEN3421) to avoid putative accumulation within cells during prolonged incubation. PAF (1 or 10 µM) was added together with gentamicin. After 6 or 24 h, supernatant was recovered for nitrite dosage and stocked at −20°C. Cells were washed and lysed with RNA lysis buffer or sterile water for 30 min at 4°C. Intracellular bacteria were enumerated by viable counts on LB agar plates. Percentage of growth was calculated as the number of intracellular bacteria at a given time point divided by the number of bacteria at *T* = 0.

### *In Vivo* Infection and Chicken Tissue Samples

Four-week-old B13/B13 histocompatible chickens were infected intratracheally with 10^9^ colony-forming units of the highly adhesive/invasive APEC strain BEN2908 in 200 µl of sterile endotoxin-free NaCl 0.9% solution. The inoculum was prepared with bacteria in the mid-log-phase of growth. The control group was inoculated with sterile NaCl 0.9% solution. Lung and liver samples were collected aseptically from each chicken 6 h post bacterial inoculation. Tissue samples for RNA extraction were snap frozen in liquid nitrogen and stocked at −80°C. For bacterial quantification, lung samples were weighed, placed in gentleMACS™ C tubes containing sterile PBS, and homogenized using a gentle MACS™ Dissociator (Miltenyi Biotec, Germany). Dilutions of lung homogenates were plated onto Drigalski agar plates supplemented with nalidixic acid (30 µg/ml) for bacterial quantification, and 1 ml was incubated in brain heart infusion for qualitative detection of *E. coli*.

### Cell Permeability Assay

Primary chAEC were seeded at a density of 5 × 10^4^ cells on fibronectin-coated Falcon^®^ Permeable Support (8.0 µm pore) PET membranes (Corning, NY, USA), placed in 24-well plates containing EC medium and cultured for 72 h at 40°C under 5% CO_2_. Then, chAEC monolayers in the upper chamber received different stimuli and, at different time points, EC growth medium containing 1 mg/ml FITC-dextran (40,000 MW) (Sigma-Aldrich, UK) was added followed by further incubation for 30 min at 40°C under 5% CO_2_. Then, 50 µl were collected from the lower chamber, and fluorescence signals relating to FITC-dextran passage from the upper to the lower chamber were measured using a GloMax^®^-Multi Detection System plate reader (Promega, UK). Cell layer permeability was expressed as fold increase in FITC-dextran fluorescence as compared to the control group (vehicle).

### Protein Homology Analysis

Protein multiple sequence alignment for PAFR and LPCAT 2 (LPCAT2) from chicken, mouse, and human were performed using ClustalW2 through Geneious R10 software (Biomatters Ltd., New Zealand). Homology analyses of transmembrane and cytoplasmic domains for PAFR or LPCAT2, including exclusive LPCAT2 enzymatic-related domains, were performed using Uniprot Blast.^2^ Predicted phosphorylation sites were identified using NetPhos 3.1 server.^3^ Only intracellular predicted sites with a score above 0.650 and conserved in the three analyzed sequences were annotated. Predicted N-glycosylation sites conserved in the three analyzed sequences were identified using NetNGlyc 1.0 server.^4^ NCBI Reference Sequence for these proteins were as follows: PAFR (*Homo sapiens*: NP_001158195, *Mus musculus*: NP_001074680, and *Gallus gallus*: XP_004947758) and LPCAT2 (*H. sapiens*: NP_060309, *M. musculus*: NP_766602, and *G. gallus*: NP_001025739).

### Statistical Analysis

Comparisons between two groups were performed using a two-tailed unpaired Student’s *t* test. Multiple groups were compared using a one-way analysis of variance followed by a Newman–Keuls multiple comparison *post hoc* test. Values for all measurements are expressed as mean ± SEM. *P* < 0.05 was considered statistically significant. Statistical analyses were performed using GraphPad Prism 5.0 (GraphPad Software, USA). Data are representative of at least two independent experiments.

## Results

### Protein Homology Analysis of PAFR and LPCAT2

Platelet-activating factor receptor is a class A rhodopsin-like GPCR. Homology analysis showed that chicken PAFR possesses protein sequence identity of 60.3 and 58.0% as compared to human or mouse PAFR protein sequences, respectively. Within transmembrane domains (TMD) 2, 3, and 7 sequence identity may reach 80–90%. Multiple sequence alignments of chicken PAFR to human and mouse counterparts revealed that these proteins have seven hydrophobic α-helical domains and conserved amino acids (or amino-acid motifs) commonly found in members of the rhodopsin family GPCRs: an asparagine (N) in the first TMD, aspartate (D) in the second and seventh TMD, a LXXXD motif in the second TMD, tryptophan (W) in the fourth TMD, conserved cysteine (C) residues in the first two extracellular loops (believed to form an S–S bridge and contribute to protein stability), a FXP motif in the sixth TMD (also found as WXP in other GPCRs), and a DPXXY motif in the seventh TMD (also found as NPXXY in other GPCRs) (Figure S1 in Supplementary Material). Importantly, in the chicken PAFR sequence the residues corresponding to the DRY motif are NRF, lacking the conserved tyrosine residue in the second intracellular loop. The DRY motif is a GPCR signature triplet sequence found downstream of the third TMD, which is believed to be involved in G-protein coupling (45–48). Chicken PAFR also contains two conserved predicted sites for N-linked glycosylation and nine threonine (T) or serine (S) residues identified as potential sites for phosphorylation by protein kinase A, G, or C (Figure S1 in Supplementary Material).

Chicken LPCAT2 homology analysis revealed a protein sequence identity of 62.5% as compared to both human and mouse LPCAT2 protein sequences. Multiple sequence analysis also showed that chicken LPCAT2 contains a large conserved cytoplasmic domain, one conserved TMD (different from human LPCAT2, which possesses three TMD) and two conserved motifs within an acyltransferase conserved region: HXXXXD and EGTC, believed to be essential for acyltransferase activity (49) (Figure S2 in Supplementary Material). Also, chicken LPCAT2 possesses a conserved putative EF-hand domain, similar to many Ca^2+^-binding proteins, which contains eight predicted Ca^2+^ binding sites (13, 49). The presence of a conserved C-terminal sequence motif KKXX suggests that LPCAT2 is likely to be localized to the ER in chickens (50) (Figure S2 in Supplementary Material).

### PAFR and LPCAT2 Are Expressed in Selected Chicken Tissues and Macrophages

**Figure 1:**
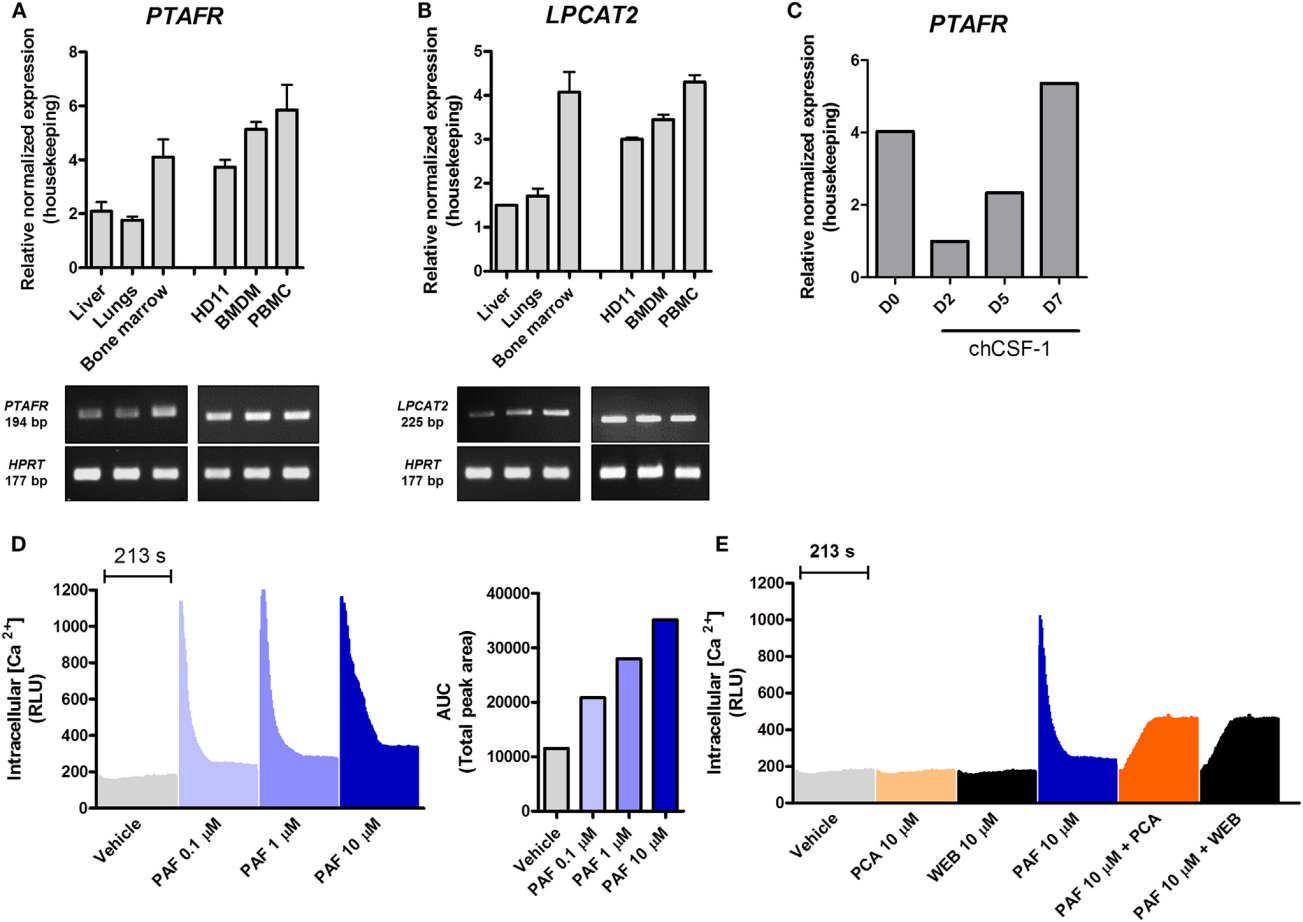
Components of the platelet-activating factor (PAF) system are expressed in chickens, and exogenous PAF induces intracellular calcium increase in chicken macrophages. **(A)** The gene expressions of PAF receptor (PAFR)/*PTAFR* and **(B)** LPCAT2/*LPCAT2* were evaluated by quantitative real-time PCR (qRT-PCR) in unstimulated chicken tissues and macrophages [HD11 macrophage-like cells, bone marrow-derived macrophages (BMDM) and peripheral blood mononuclear cells (PBMC)]. Amplicon size (in base pairs, bp) was confirmed in 2% agarose gels. **(C)** PAFR/*PTAFR* gene expression was evaluated by qRT-PCR in purified bone marrow cells following chCSF-1 medium complementation for BMDM differentiation from days 0 to 7. Data are expressed as relative normalized expression (as compared to two housekeeping genes). Values are mean ± SEM. In **(D,E)** the HD11 cell line was stimulated with PAF and/or PAFR antagonists (PCA 4248 and WEB 2086), and the increase in intracellular calcium signal was recorded over time (216 s, 3 s intervals) using a fluorescent probe. RLU, relative luminescence units; AUC, area under the curve.

To gain further insights into PAF function in chickens, we started out to evaluate gene expression for PAFR/*PTAFR* and LPCAT2/*LPCAT2* in selected healthy chicken tissues (liver, lungs, and bone marrow) and unstimulated macrophages/monocytes by qRT-PCR. We found that PAFR is abundantly expressed in chicken tissues as compared to housekeeping genes, with a twofold higher expression found in the bone marrow as compared to other organs (Figure 1A). In monocytes/macrophages, PAFR is expressed at similar levels, suggesting that a cell line (HD11), BMDM, and PBMC consistently express PAFR (Figure 1A). LPCAT2 gene expression followed the same pattern as for PAFR expression, with LPCAT2 being highly expressed in the bone marrow as compared to other organs (Figure 1B). In monocytes and macrophages, LPCAT2 was found to be consistently expressed, which suggests that chicken macrophages are able to produce PAF (Figure 1B). Interestingly, following chCSF-1 treatment to promote macrophage differentiation, bone marrow mononuclear cells quickly showed downregulation of PAFR expression (threefold), which returned to expected (BMDM) levels with the cells getting reprogrammed to differentiate into macrophages (Figure 1C).

### PAF Induces Intracellular Calcium Increase in Chicken Macrophages

We next investigated whether chicken macrophages were responsive to exogenous PAF (PAF C16). Intracellular calcium mobilization is a *bona fide* parameter to evaluate early events following GPCR binding and activation. HD11 macrophages were stimulated with PAF (0.1–10 µM), and intracellular calcium mobilization was measured using a fluorescent calcium probe. PAF induced a quick concentration-dependent increase in intracellular calcium signal which returned to levels close to vehicle-treated wells after 213 s (Figure 1D). While not interfering with intracellular calcium signal alone, pretreatment with canonical PAFR antagonists PCA 4248 or WEB 2086 efficiently blocked intracellular calcium mobilization in chicken macrophages upon exposure to PAF (10 µM) (Figure 1E). This suggests that PAF seems to act in a specific receptor (PAFR)-mediated fashion in chicken macrophages as in mammalian cells.

### PAF Binds to PAFR in Chicken Macrophages

**Figure 2:**
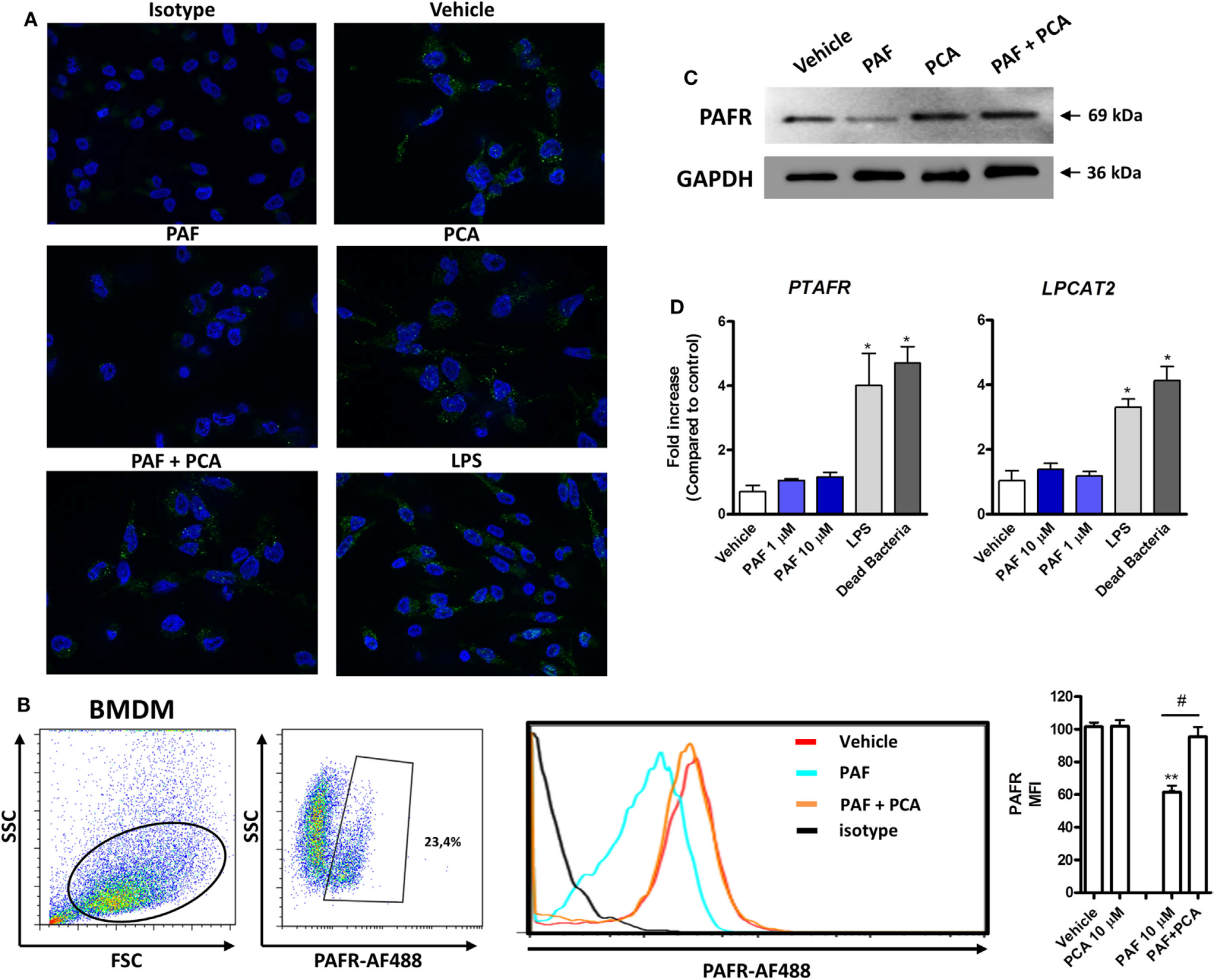
Platelet-activating factor receptor (PAFR) protein expression in chicken primary macrophages can be modulated by exogenous platelet-activating factor (PAF) or lipopolysaccharide (LPS). **(A)** Immunofluorescence (IF) analysis of PAFR protein expression using an anti-PAFR polyclonal antibody, or isotype control, in B13/B13 histocompatible chicken bone marrow-derived macrophages (BMDM). BMDM were stimulated with PAF (10 µM), *Escherichia coli* LPS (10 ng/ml) or PCA 4248 (10 µM) for 2 h before IF analysis. PAFR expression is shown in green (Alexa-Fluor 488-conjugated secondary antibody) and the nucleus in blue (DAPI staining). **(B)** Representative flow cytometry dot plots showing gating strategy for PAFR expression analysis in BMDM and histogram showing PAFR expression upon BMDM stimulation for 2 h with PAF and/or PCA 4248 as compared to isotype control. MFI, mean fluorescence intensity. **(C)** Western blot analysis of PAFR expression in BMDM whole cell lysates following different treatments for 2 h. GAPDH was used as loading control. Target protein size is indicated with black arrows. **(D)** PAFR/*PTAFR* and LPCAT2/*LPCAT2* gene expression analysis in BMDM stimulated with PAF, LPS, or dead bacteria (10 multiplicity of infection) for 6 h. Data are expressed as relative normalized expression (as compared to vehicle control group). IF images were captured using an Axiovert 200 M inverted epi-fluorescence microscope equipped with an EC Plan-Neofluar 40 × /1.3 oil/Dic objective (ApoTome system, Zeiss). Values are mean ± SEM. *P < 0.05, **P < 0.01 when compared to negative (vehicle) control group. ^#^*P* < 0.05 when compared to positive control groups.

To characterize PAFR protein expression and modulation by exogenous PAF in chicken primary macrophages, IF, WB, and flow cytometry (FACS) analyses using a commercially available antibody predicted to cross-react with different species were performed. PAFR was clearly expressed in chicken BMDM at protein level (Figure 2A, upper panels) as was validated with the isotype control. Interestingly, the addition of PAF (10 µM) for 2 h led to PAFR downregulation, as seen by the loss of fluorescent signal within stained macrophages (Figure 2A, middle panels). Following PAFR activation, cells become rapidly desensitized, and this refractory state is dependent on PAFR phosphorylation, internalization, and downregulation (51). PAFR antagonist PCA 4248 did not promote PAFR downregulation alone, but abrogated PAFR downregulation induced by exogenous PAF (Figure 2A, middle and lower panels). This phenomenon was confirmed by FACS analysis, where around 23% of BMDM expressed PAFR and the addition of exogenous PAF for 2 h led to receptor downregulation as seen through changes in PAFR mean fluorescence intensity (Figure 2B). Also, WB analysis confirmed that PAFR is partially degraded upon PAF binding and that PCA 4248 blocked this phenomenon (Figure 2C). Altogether, these data revealed that PAFR is expressed at the protein level and can be downregulated and partially degraded by PAF in chicken macrophages.

In addition to this physiological phenomenon, we observed that PAFR gene expression is increased upon *E. coli* LPS or DB stimulation, but not upon exogenous PAF treatment, for 6 h as compared to vehicle-treated group (Figure 2D), as was assessed by IF analysis (Figure 2A, lower right panel). LPCAT2 gene expression was also upregulated by these pro-inflammatory bacterial stimuli, but not by exogenous PAF, which suggests that bacteria-associated molecular patterns are positive signals for the upregulation of PAFR and LPCAT2 genes, the latter being important for the metabolism of PAF during inflammation.

### PAF Promotes Increased Phagocytosis and ROS Release by Chicken Macrophages

**Figure 3:**
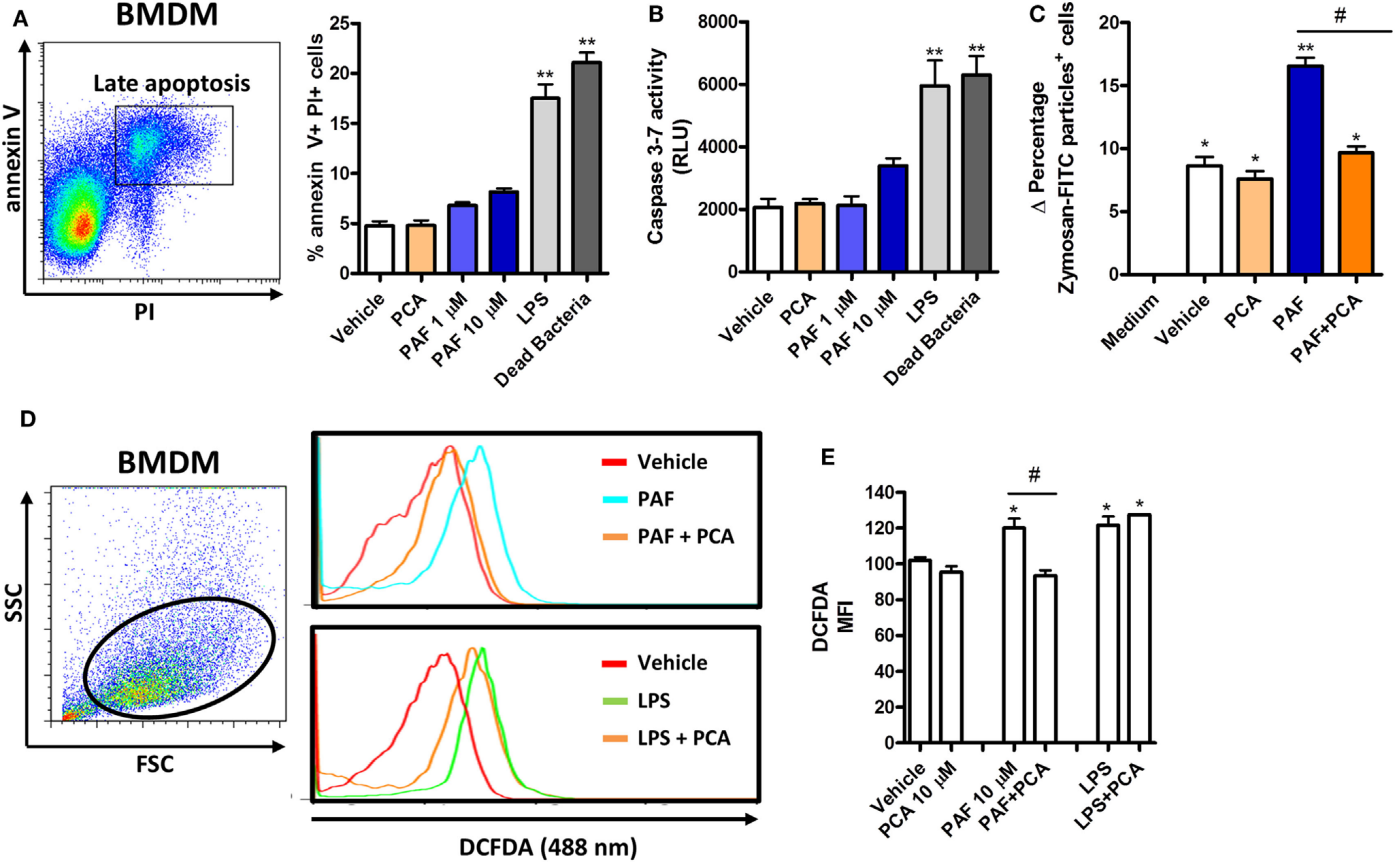
Exogenous platelet-activating factor (PAF) treatment does not impact cell viability and induces reactive oxygen species (ROS) release by chicken primary macrophages. **(A)** Representative flow cytometry dot plot and bar graph showing gating strategy for annexin V and propidium iodide (PI) expression analysis in bone marrow-derived macrophages (BMDM) from B13/B13 histocompatible chicken and percentage of double-positive stained cells, respectively, following treatment with PCA 4248 (10 µM), PAF (1 or 10 µM), *Escherichia coli* lipopolysaccharide (LPS) (10 ng/ml) or dead bacteria (10 multiplicity of infection) for 6 h. **(B)** Caspase 3–7 activity in BMDM following exposure to different stimuli for 6 h. RLU: relative luminescence units. **(C)** Delta percentage of BMDM containing zymosan-fluorescein isothiocyanate particles following different stimuli for 1 h as an index of phagocytosis (delta calculated after incubation at 4°C and 40°C). **(D)** Representative flow cytometry dot plot and histograms showing ROS release by BMDM, as determined by intracellular DCFDA fluorescence intensity, following different stimuli for 2 h. **(E)** Bar graph showing the mean fluorescence intensity (MFI) of DCFDA-positive BMDM following different stimuli for 2 h. Values are mean ± SEM. *P < 0.05, **P < 0.01 when compared to negative (vehicle or medium) control groups. ^#^*P* < 0.05 when compared to positive control groups.

To further address the functional role of PAF in chicken primary macrophages, we first assessed whether exogenous PAF were cytotoxic to these cells. We showed that PAF (1 and 10 µM) was not cytotoxic to chicken BMDM after 6 h stimulation as evaluated through annexin V/PI double-staining strategy (Figure 3A) and caspase 3–7 activation assay (Figure 3B), both canonical markers for early and late cell apoptosis. On the other hand, *E. coli* LPS and DB were cytotoxic to chicken BMDM within the same experimental settings.

We next asked whether PAF could enhance BMDM phagocytosis of fluorescent zymosan beads. We observed that PAF (10 µM) treatment for 1 h significantly enhanced chicken macrophage phagocytosis and that PAFR antagonist PCA 4248 abrogated this phenomenon (Figure 3C).

In another biologically relevant assay, we assessed whether PAF induces ROS release by chicken macrophages. Using a fluorescent probe, we showed that both PAF (10 µM) and *E. coli* LPS stimulation for 2 h promoted enhanced ROS release by chicken BMDM. However, PAFR antagonist PCA 4248 reverted ROS release only in PAF-treated BMDM, revealing that ROS release is induced by PAF in a PAFR-dependent manner (Figures 3D,E). Altogether these data suggest that PAF promotes two acute macrophage-related inflammatory events in a PAFR-dependent fashion and independent of any cytotoxic effect in chicken macrophages.

### PAF Contributes to LPS Pro-inflammatory Response in Chicken Macrophages

**Figure 4:**
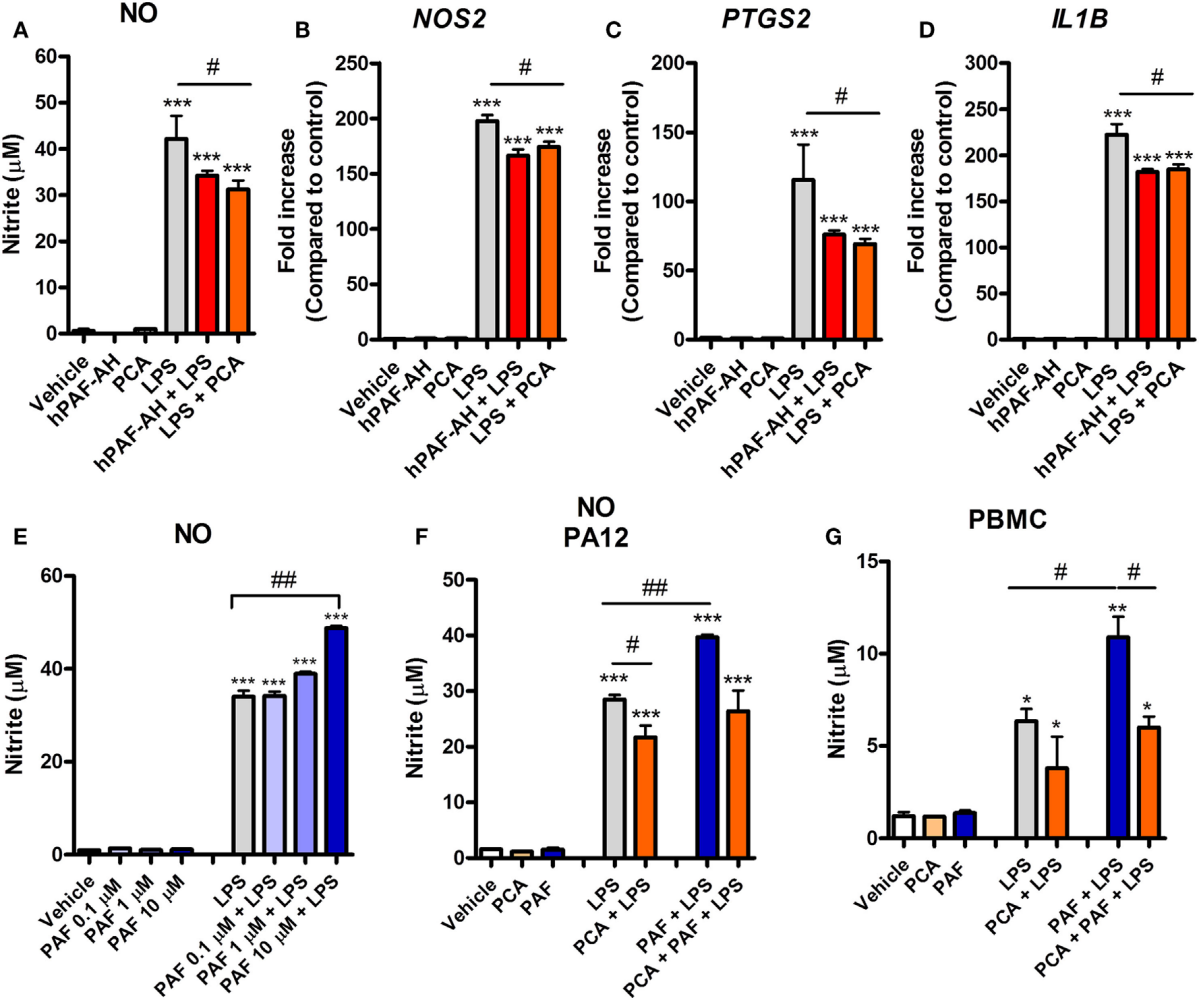
Endogenous platelet-activating factor (PAF) contributes to the pro-inflammatory response elicited by lipopolysaccharide (LPS) and exogenous PAF potentiates LPS-induced inflammation in chicken primary macrophages. After stimulation with *E. coli* LPS (10 ng/ml) in the presence or absence of PCA 4248 (10 µM) or human PAF-acetylhydrolase (PAF-AH) (10 µg/ml) for 6 h, B13/B13 histocompatible chicken-derived bone marrow-derived macrophage (BMDM) supernatants were assessed for the presence of **(A)** nitric oxide (NO), and cell lysates were used to analyze gene expression of **(B)** iNOS/*NOS2*, **(C)** COX-2/*PTGS2*, and **(D)** IL-1β/*IL1B* by quantitative real-time PCR. Data are expressed as relative normalized expression (as compared to vehicle control group). **(E)** PAF (0.1–10 µM) alone or together with LPS was added to BMDM, and NO production in the supernatants was assessed after 6 h. **(F)** NO production by BMDM derived from the bone marrow of the outbred PA12 chickens following exposure to PAF (10 µM) and/or LPS in the presence or absence of PCA 4248 (10 µM) for 6 h. **(G)** NO production by peripheral blood mononuclear cells (PBMC) from B13/B13 histocompatible chickens following exposure to PAF (10 µM) and/or LPS in the presence or absence of PCA 4248 (10 µM) for 6 h. Values are mean ± SEM. *P < 0.05, **P < 0.01, and ***P < 0.001 when compared to negative (vehicle) control groups. ^#^*P* < 0.05 and ^##^*P* < 0.01 when compared to positive control groups.

A previous study showed that rabbit alveolar macrophages cytokine production in response to endotoxin is PAF dependent (21). We therefore asked whether endogenous PAF production contributes to the LPS-induced pro-inflammatory response in chicken macrophages at 6 h. We observed that PAFR blockade with PCA 4248 or the addition of recombinant human PAF-AH (which degrades extracellular PAF) together with LPS led to significantly reduced NO production and reduced expression of pro-inflammatory genes such as iNOS/*NOS2*, COX-2/*PTGS2*, and IL-1β/*IL1B* as compared to groups treated with LPS alone (Figures 4A–D). These data suggest that PAF produced by chicken macrophages upon LPS stimulation contributes to the overall pro-inflammatory effects of LPS *via* PAFR.

### PAF Amplifies the Inflammatory Response Induced by *E. coli* LPS

We next asked whether PAF could directly induce the production or expression of inflammatory mediators by chicken macrophages. Starting with NO, we showed that exogenous PAF (0.1–10 µM) alone is not able to induce NO production by chicken BMDM, whereas LPS is a potent NO production inducer (Figure 4E). However, when added together with LPS, PAF (10 µM) promoted a significant increase in LPS-induced NO production by chicken macrophages as compared to groups receiving LPS alone (Figure 4E). We also demonstrated this phenomenon in BMDM from another chicken line (PA12) (Figure 4F) and in PBMC of the B13 chicken line (Figure 4G), suggesting that PAF potentiation of LPS-induced NO production is biologically consistent among chicken macrophages/monocytes. In these latter cells, we also observed the aforementioned contribution of endogenous PAF to LPS-induced production of pro-inflammatory mediators (here NO) through PAFR blockade with PCA 4248 upon LPS-stimulation (Figures 4F,G), thus corroborating our previous findings.

### PAF Potentiation Effects Are Mediated by PI3K and CaMK II Signaling Pathways

**Figure 5:**
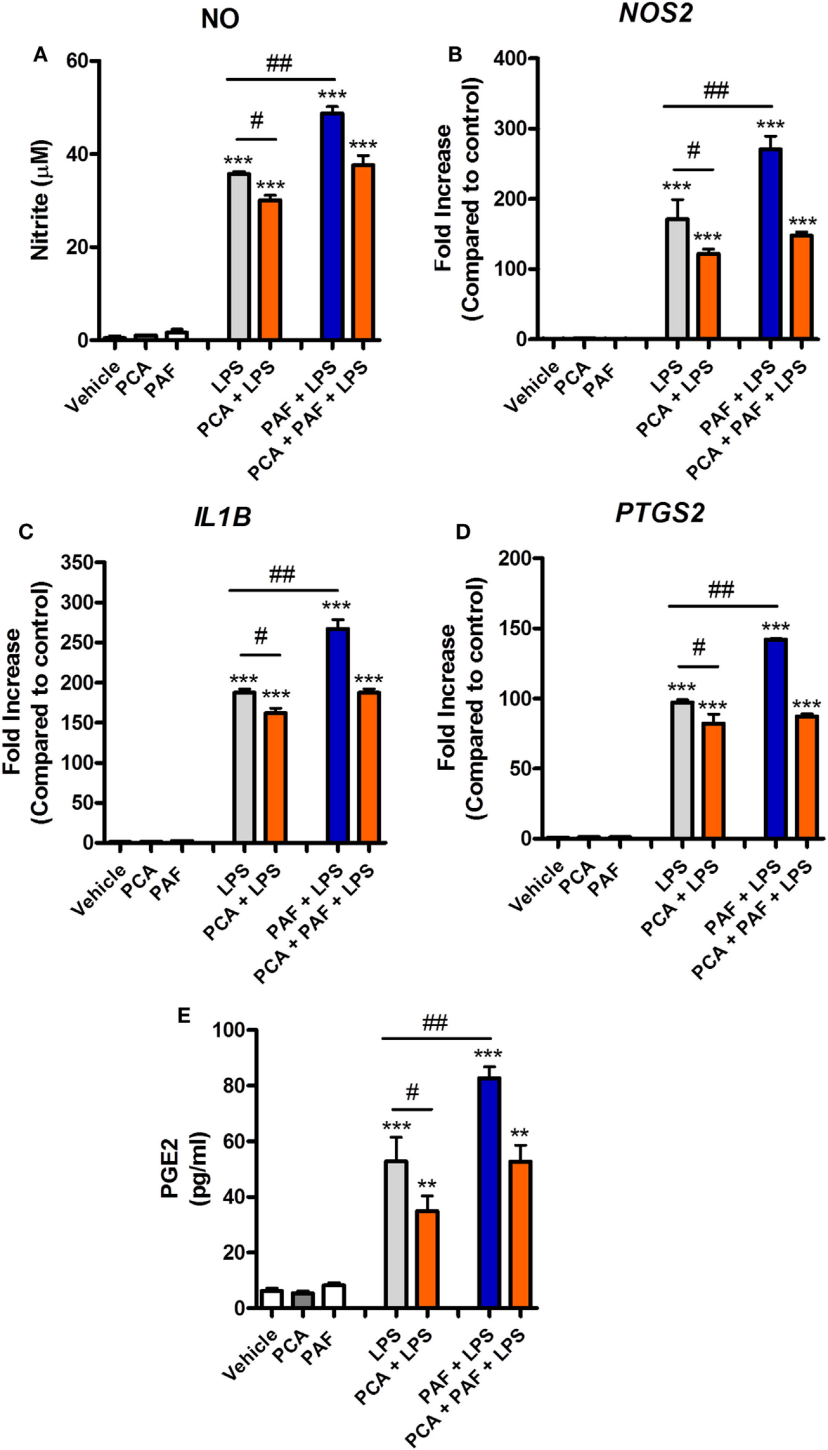
Exogenous platelet-activating factor (PAF) potentiates the pro-inflammatory response elicited by lipopolysaccharide (LPS) in chicken primary macrophages in a PAF receptor (PAFR)-dependent manner. After 6 h stimulation with PAF (10 µM) and/or *E. coli* LPS (10 ng/ml) in the presence or absence of PAFR antagonist PCA 4248 (10 µM), B13/B13 histocompatible chicken-derived bone marrow-derived macrophage (BMDM) supernatants were assessed for the presence of **(A)** nitric oxide, and cell lysates were used to analyze gene expression of **(B)** iNOS/*NOS2*, **(C)** IL-1β/*IL1B*, and **(D)** COX-2/*PTGS2* by quantitative real-time PCR. Data are expressed as relative normalized expression (as compared to vehicle control group). **(E)** Prostaglandin E2 dosage by mass spectrometry in BMDM supernatants following the same aforementioned stimuli for 6 h. Values are mean ± SEM. **P < 0.01 and ***P < 0.001 when compared to negative (vehicle) control groups. ^#^*P* < 0.05 and ^##^*P* < 0.01 when compared to positive control groups.

To gain further insight into the mechanisms through which PAF potentiates the pro-inflammatory effects of LPS, we first confirmed that exogenous PAF (10 µM) not only potentiates the production of NO induced by *E. coli* LPS by chicken BMDM but also enhances iNOS/*NOS2*, IL-1β/*IL1B*, and COX-2/*PTGS2* gene expression, without being able to induce the expression of these genes alone (Figures 5A–D). In line with COX-2/*PTGS2* upregulation, exogenous PAF potentiates PGE2 (which is metabolized by COX-2) production by chicken macrophages upon LPS stimulation, despite its inability to directly induce the production of this eicosanoid (Figure 5E).

**Figure 6:**
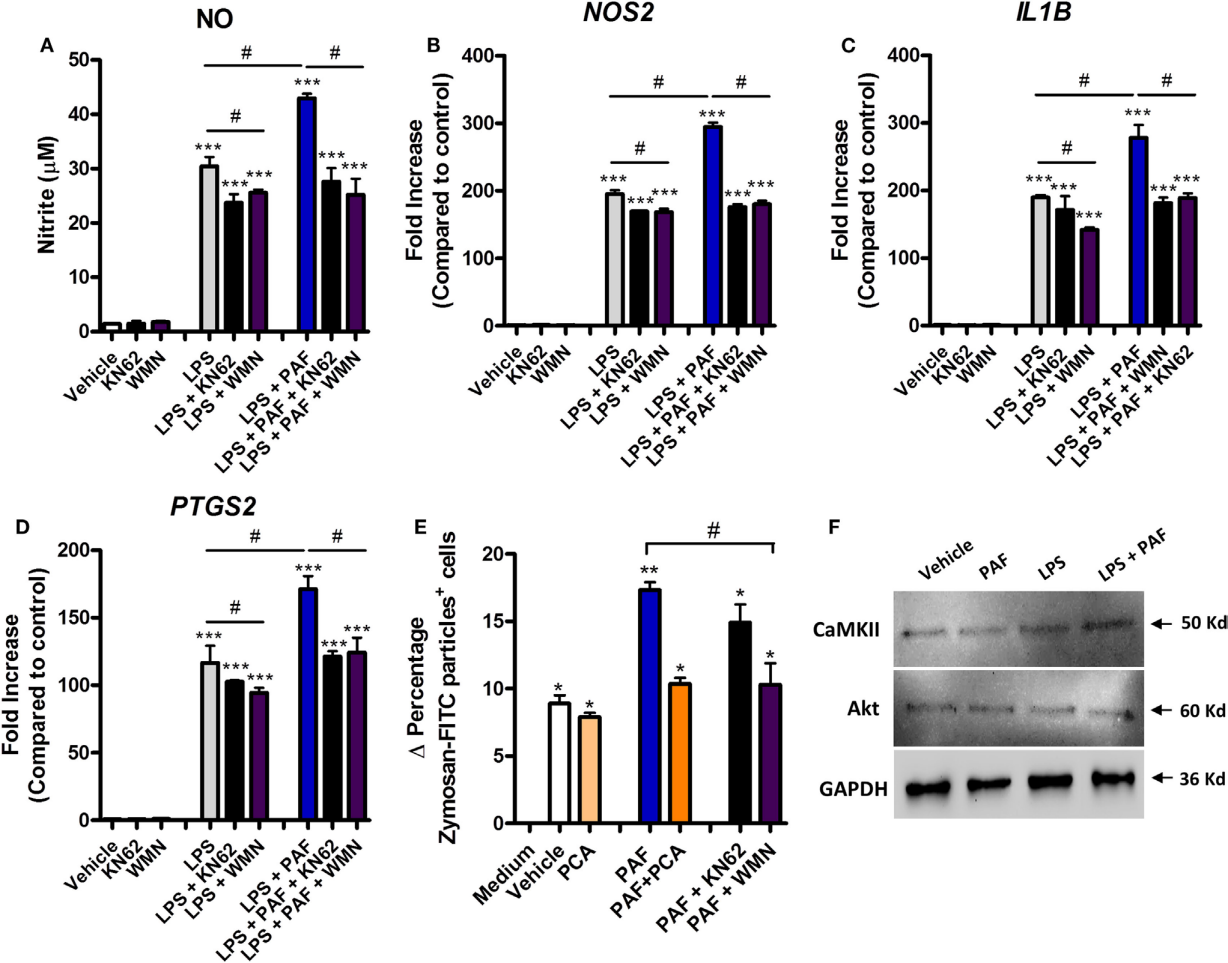
The potentiation effects of platelet-activating factor (PAF) on lipopolysaccharide (LPS)-induced pro-inflammatory response in chicken primary macrophages are partially dependent on CaM KII- and phosphatidylinositol 3-kinase (PI3K)/Akt-mediated signaling pathways. After 6 h stimulation with PAF (10 µM) and/or *Escherichia coli* LPS (10 ng/ml) in the presence or absence of PI3K antagonist wortmannin (WMN, 200 nM) or CaM KII antagonist (KN62, 1 µM), B13/B13 histocompatible chicken-derived bone marrow-derived macrophage (BMDM) supernatants were assessed for the presence of **(A)** nitric oxide, and cell lysates were used to analyze gene expression of **(B)** iNOS/*NOS2*, **(C)** IL-1β/*IL1B*, and **(D)** COX-2/*PTGS2* by quantitative real-time PCR. Data are expressed as relative normalized expression (as compared to vehicle control group). **(E)** Delta percentage of BMDM containing zymosan-fluorescein isothiocyanate particles following different stimuli for 1 h as an index of phagocytosis (delta calculated after incubation at 4°C and 40°C). **(F)** Western blot analysis of CaM KII and Akt expression in BMDM whole cell lysates following different treatments for 6 h. GAPDH was used as loading control. Target protein size is indicated with black arrows. Values are mean ± SEM. *P < 0.05, **P < 0.01 and ***P < 0.001 when compared to negative (vehicle or medium) control groups. ^#^*P* < 0.05 when compared to positive control groups.

Signaling pathways such as those mediated by PI3K, calcium CaMK II, and mitogen-activated protein kinase (MAPK) have been shown to be involved in mediating the effects of PAF in mammalian leukocytes (52, 53). Pretreatment with the PI3K inhibitor WMN (200 nM) or CaMK II inhibitor KN62 (1 µM) significantly diminished the potentiation effects of exogenous PAF on LPS-induced upregulation of NO production and iNOS/*NOS2*, IL-1β/*IL1B*, and COX-2/*PTGS2* gene expression in chicken BMDM (Figures 6A–D). PI3K inhibition, but not CamK II inhibition, also abrogated exogenous PAF potentiation of zymosan beads phagocytosis by chicken macrophages, leading to a similar level than that observed when PAF signaling is blocked with PAFR antagonist PCA 4248 (Figure 6E). Importantly, these inhibitors showed mild yet statistically significant effects on LPS-induced NO production and upregulation of pro-inflammatory genes expression (Figures 6A–D), in a magnitude that does not appear to interfere with their inhibitory action on PAFR signaling and its potentiation effects as suggested. We did not observe any statistically significant effects in the aforementioned parameters when the p38 MAPK-mediated signaling pathway was blocked using the specific inhibitor SB 203580 (data not shown).

We also confirmed that Akt (which is phosphorylated by PI3K) and CaMK II are expressed in chicken BMDM at protein levels (Figure 6F), although we could not determine the presence of PI3K due to the unavailability of validated commercial antibodies known to recognize this enzyme in chickens. We therefore presume that PAF exerts its pro-phagocytic and potentiating effects over LPS-induced pro-inflammatory responses through PI3K/Akt- and/or CaMK II-mediated intracellular signaling pathways.

### PAF Contributes to Intracellular Bacteria Killing

**Figure 7:**
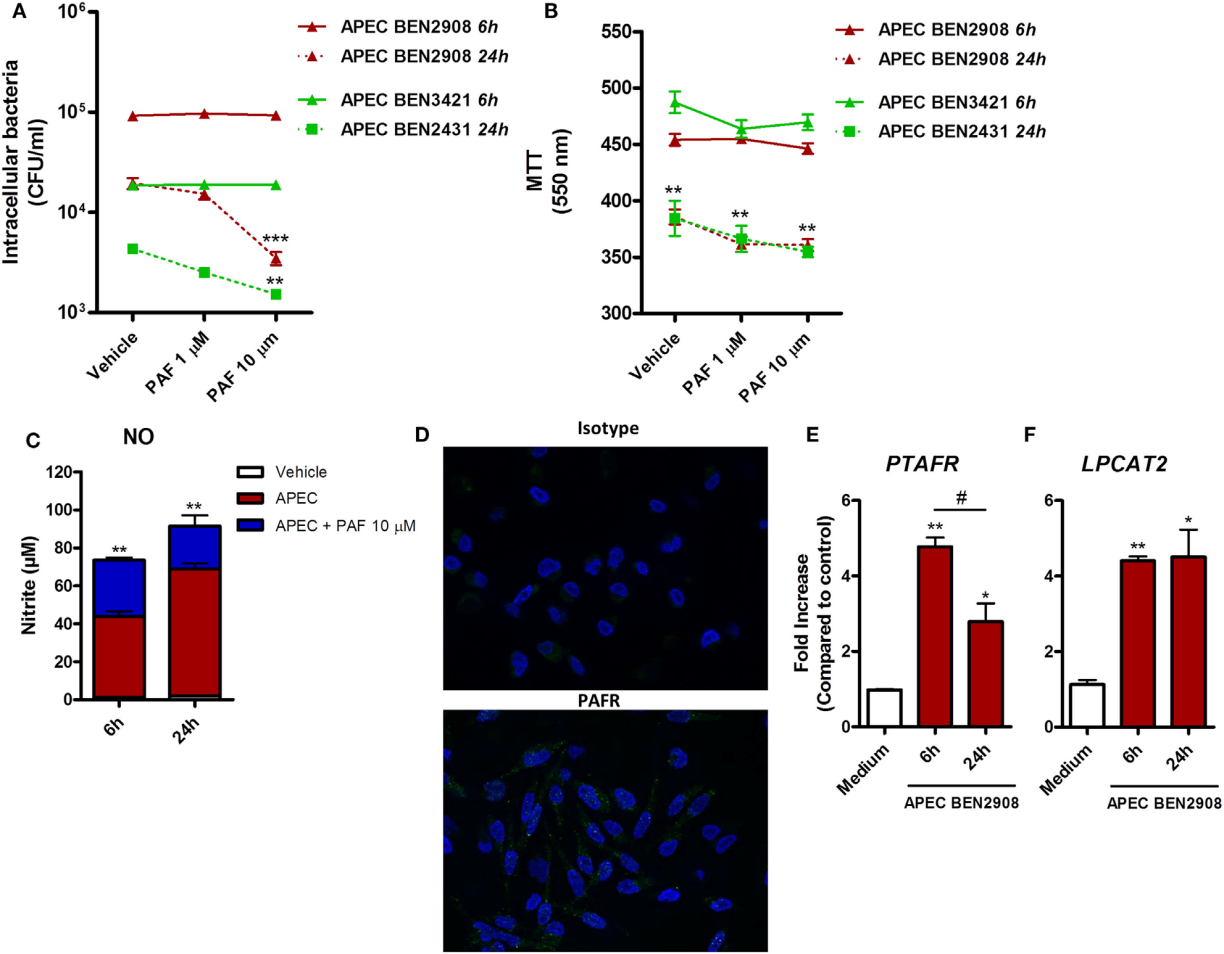
Exogenous platelet-activating factor (PAF) treatment favors intracellular bacteria killing and amplifies the inflammatory response in infected chicken macrophages. **(A)** HD11 macrophage-like cells were infected with 10 multiplicity of infection (MOI) of avian pathogenic *Escherichia coli* (APEC) strains BEN2908 or BEN3421 (both O2 serotype strains) followed by treatment with PAF (1 or 10 µM) for 6 or 24 h. Intracellular bacterial load was evaluated through colony-forming unit counts. **(B)** Cell viability was evaluated through a colorimetric assay based on methylthiazoletetrazolium bromide cellular metabolism, following the same *in vitro* infection protocol. Data are expressed in optical density at 550 nm. **(C)** Nitric oxide production in the supernatants of HD11 cells following infection with 10 MOI of APEC BEN2908 in the presence or absence of PAF. **(D)** Immunofluorescence (IF) analysis of PAF receptor (PAFR) protein expression using an anti-PAFR polyclonal antibody, or isotype control, in HD11 chicken macrophage cell line. PAFR expression is shown in green (Alexa-Fluor 488-conjugated secondary antibody) and the nucleus in blue (DAPI immunostaining). IF images were captured using an Axiovert 200 M inverted epi-fluorescence microscope equipped with an EC Plan-Neofluar 40 × /1.3 oil/Dic objective (ApoTome system, Zeiss). **(E)** PAFR/*PTAFR* and **(F)** LPCAT2/*LPCAT2* gene expression analysis in HD11 cells following infection with 10 MOI of the APEC BEN2908 strain. Quantitative real-time PCR data are expressed as relative normalized expression (as compared to vehicle control group). Values are mean ± SEM. *P < 0.05, **P < 0.01, and ***P < 0.001 when compared to negative (vehicle or medium) control groups. ^#^*P* < 0.05 when compared to positive control groups.

In addition to its pro-inflammatory effects in chicken macrophages, we assessed whether exogenous PAF could promote increased intracellular bacteria killing in macrophages infected with two phenotypically different strains of APEC. Although both strains belong to the O2 serotype, the highly adhesive/invasive strain BEN2908 was able to better infect HD11 macrophages (1 log difference), as compared to the BEN3421 strain at the same MOI and at the same time points (Figure 7A). The addition of exogenous PAF following APEC infection for 6 h did not promote increased intracellular APEC killing as compared to infected groups receiving vehicle only. However, at 24 h, PAF (10 µM) was able to significantly reduce intracellular bacterial load, notably for the highly virulent BEN2908 strain (Figure 7A). This phenomenon was not related to increased PAF-driven macrophage cytotoxicity since infected cells receiving PAF showed the same cell death level as infected cells receiving vehicle at both 6 and 24 h, as was determined by the MTT assay (Figure 7B). As observed before with LPS, the addition of exogenous PAF to APEC infected macrophages led to time-dependent increase in NO production as compared to untreated infected macrophages (Figure 7C), which suggests that PAF also amplifies the inflammatory response in APEC-infected macrophages. We also confirmed by IF that PAFR is expressed at protein level in HD11 macrophage cell line (Figure 7D). In addition, APEC infected macrophages showed increased upregulation of PAFR/*PTAFR* and LPCAT2/*LPCAT2* gene expression (Figures 7E,F). Overall, we showed that PAF is able to promote increased intracellular bacteria killing together with an increased pro-inflammatory response. Also, we suggest that APEC infection is likely to promote increased PAF signaling and production by chicken macrophages.

### PAFR and LPCAT2 Expression Are Upregulated during Pulmonary Colibacillosis

**Figure 8:**
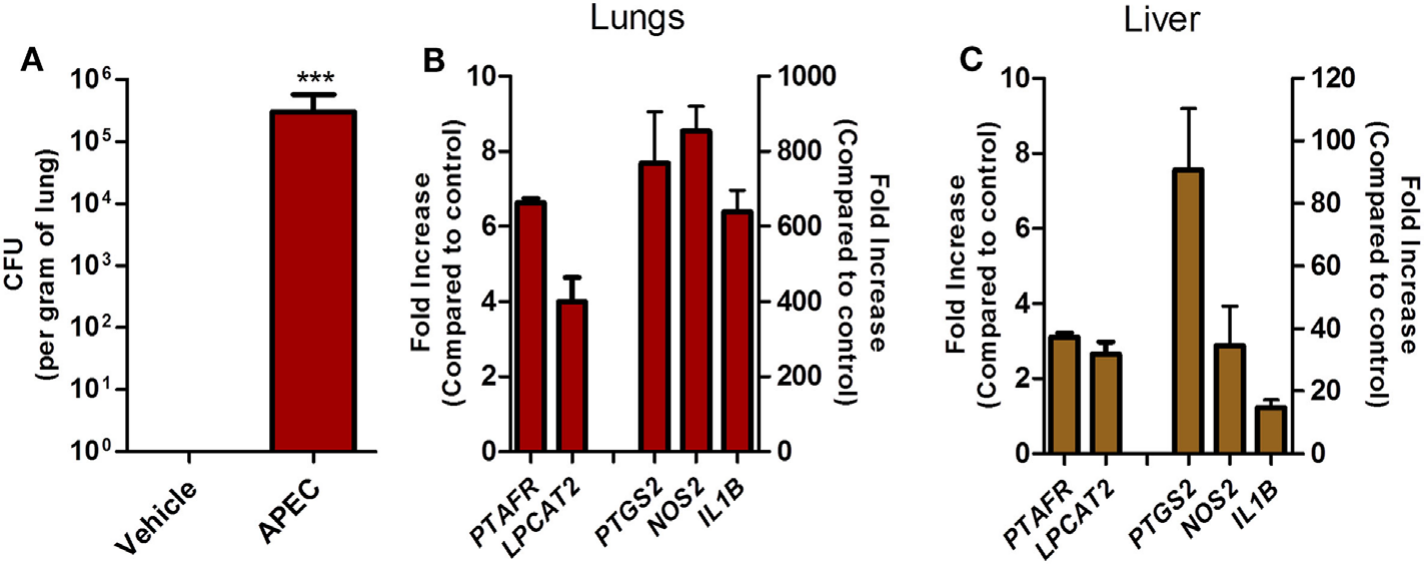
Overexpression of platelet-activating factor receptor (PAFR) and LPCAT2 is correlated with increased pro-inflammatory gene expression in chicken organs during pulmonary colibacillosis. **(A)** Bacterial load in the lungs of B13/B13 histocompatible white leghorn chickens at 6 h following intratracheal infection with 10^9^ colony forming units of APEC BEN2908 (O2 serotype) strain. **(B)** Lung or **(C)** liver samples from APEC BEN2908-infected chickens were analyzed for PAFR/*PTAFR*, LPCAT2/*LPCAT2*, COX-2/*PTGS2*, iNOS/*NOS2*, and IL-1β/*IL1B g*ene expression by quantitative real-time PCR. Data are expressed as relative normalized expression (as compared to vehicle control group). Values are mean ± SEM. ***P < 0.001 when compared to negative (vehicle) control group. Sample size was six animals per group.

To follow-up on these findings in a more relevant experimental system, we assessed whether PAFR and LPCAT2 genes are overexpressed in chickens during experimental pulmonary colibacillosis. After 6 h following APEC BEN2908 intratracheal instillation (10^9^ bacteria/animal), chickens presented around 10^6^ bacteria in the lungs (Figure 8A). We next compared the expression of acute pro-inflammatory mediators with PAFR and LPCAT2 expression in chicken lungs and liver, two organs where typical colibacillosis lesions are known to develop. We observed that in both the lungs (Figure 8B) and liver (Figure 8C), the gene expressions of PAFR/*PTAFR* and LPCAT2/*LPCAT2* are upregulated together with pro-inflammatory gene expression such as COX-2/*PTGS2*, iNOS/*NOS2*, and IL-1β/*IL1B* as compared to vehicle-instilled animals. These data suggest that during pulmonary colibacillosis, genes involved in PAF signaling, and metabolism are overexpressed concomitantly to genes related to the acute inflammatory response, which is in agreement with the data found in APEC-infected macrophages.

### PAF Contributes to Inflammation and Cell Permeability in Chicken Endothelial Cells

**Figure 9:**
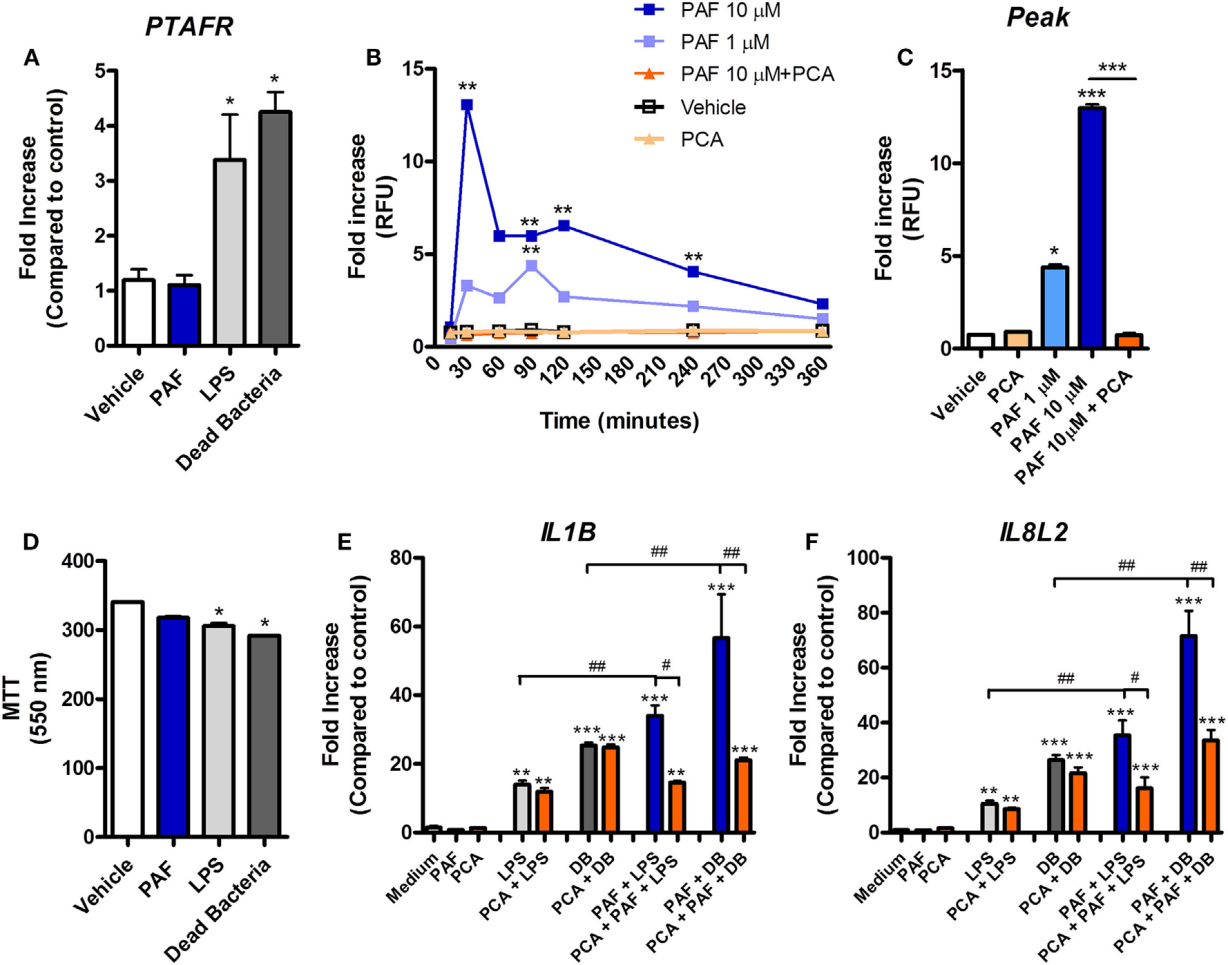
Exogenous platelet-activating factor (PAF) contributes to chicken endothelial cell dysfunction and increased pro-inflammatory response to bacteria-associated molecular patterns. **(A)** PAF receptor (PAFR)/*PTAFR* gene expression, as evaluated by quantitative real-time PCR (qRT-PCR) analysis, in chicken aortic endothelial cells (chAEC) from PA12 outbred chickens following treatment with PAF (10 µM), lipopolysaccharide (LPS) (10 ng/ml), or dead bacteria (DB, 10 multiplicity of infection) for 6 h. **(B)** Fluorescein isothiocyanate (FITC)-dextran relative fluorescence units (RFU), as an index of chAEC monolayer permeability in a transwell system, following treatment with PAF (1 or 10 µM) in the presence or absence of PCA 4248 (10 µM). **(C)** FITC-dextran RFU in the same transwell system at 30 min post chAEC treatment with PAF in the presence or absence of PCA 4248. Data are expressed as fold increase as compared to vehicle control group. **(D)** chAEC viability, as evaluated through a colorimetric assay based on methylthiazoletetrazolium bromide cellular metabolism, following treatment with PAF, LPS, or DB for 6 h. Data are expressed in optical density at 550 nm. **(E)** IL-1β/*IL1B* and **(F)** CXCLi2/*IL8L2* gene expression in chAEC following treatment with PAF, LPS, and DB for 6 h in the presence or absence of PCA 4248. qRT-PCR data are expressed as relative normalized expression (as compared to vehicle control group). Values are mean ± SEM. *P < 0.05, **P < 0.01, and ***P < 0.001 when compared to negative (vehicle) control groups. ^#^*P* < 0.05, ^##^*P* < 0.01, and ^###^*P* < 0.001 when compared to positive control groups.

**Figure 10:**
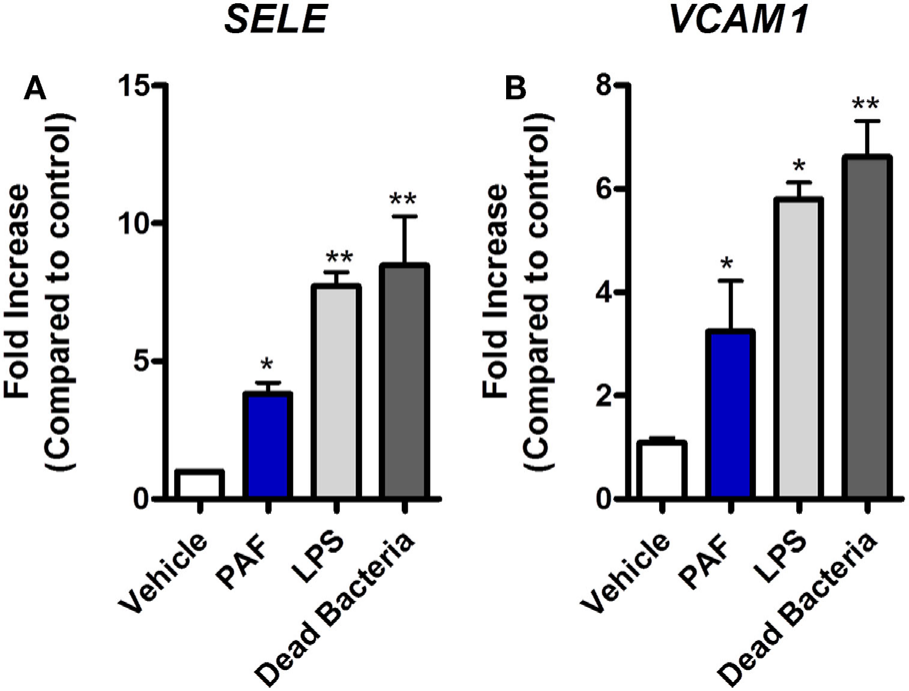
Exogenous platelet-activating factor receptor (PAF) directly promotes upregulation of adhesion molecules gene expression in chicken endothelial cells. **(A)** E-selectin/*SELE* and **(B)** VCAM-1/*VCAM1* gene expression in chicken aortic endothelial cells (chAEC) following treatment with PAF (10 µM), lipopolysaccharide (LPS) (10 ng/ml), or dead bacteria (10 multiplicity of infection) for 6 h. Quantitative real-time PCR data are expressed as relative normalized expression (as compared to vehicle control group). Values are mean ± SEM. *P < 0.05 and **P < 0.01 when compared to negative (vehicle) control groups.

Platelet-activating factor contribution to vascular physiopathology, such as increased vascular permeability and leukocyte adhesion, is well described in mammals (5, 8). Using a novel chicken endothelial cell line (chAEC), we aimed at addressing PAF contribution to increased EC permeability and inflammatory response in chickens. We first showed that PAFR/*PTAFR* is expressed in chAEC, and its expression is upregulated by LPS or DB (Figure 9A). Next, using a transwell system, we demonstrated that exogenous PAF induced increased EC permeability as assessed by FITC-dextran leakage (Figure 9B). A quick phenomenon that peaked at 30 min following exposure to PAF (10 µM) and lasted for up to 4 h (Figures 9B,C). Blockade of PAFR with PCA 4248 abrogated FITC-dextran leakage (Figures 9B,C). EC permeability was not related to PAF cytotoxic effects on chAEC as assessed by the MTT assay at 6 h (Figure 9D). As observed for macrophages, exogenous PAF treatment for 6 h did not induce pro-inflammatory gene expression alone but potentiated the pro-inflammatory effects of *E. coli* LPS or DB as found for IL-1β/*IL1B* and CXCLi2/*IL8L2* (a heterophil-recruiting chemokine) gene expression (Figures 9E,F). Blockade of PAFR with PCA 4248 reduced this potentiation to levels seen for LPS or DB alone (Figures 9E,F). Interestingly, exogenous PAF treatment alone upregulated E-selectin/*SELE* and VCAM-1/*VCAM1* gene expression in chAEC to levels close to those found for LPS or DB (Figure 10). Altogether, these data reveal that PAF is active in chicken endothelial cells and promotes increased cell permeability, potentiates bacterial-derived inflammatory responses, and may indirectly contribute to leukocyte adhesion to the chicken endothelium.

## Discussion

The physiological and pathological roles of lipid mediators in livestock animals, such as the chicken, are often neglected and little is known on their presence and mechanisms of action. Here, we provide novel insights into the function of PAF as a pro-inflammatory mediator in chickens and the mechanism through which PAF contributes to chicken macrophage activation and responses to bacterial LPS. In addition, PAF contributes to intracellular bacterial killing and EC dysfunction, two phenomena that may be correlated with the overexpression of PAFR and LPCAT2 in organs of chickens presenting colibacillosis. Overall, we provided evidence that PAF may be an early orchestrator of the inflammatory response to pathogenic bacteria in chickens.

Protein homology analysis of chicken PAFR revealed that this receptor shares several potentially functional features with its mammalian counterparts (e.g., structure, N-glycosylation, and phosphorylation sites), all important to effective PAFR signaling (8, 51). In addition, we showed that PAF is able to activate chicken macrophage PAFR, and canonical PAFR antagonists, largely tested in mammalian systems (52, 54, 55), were able to abrogate intracellular calcium increase, ROS release, and phagocytosis induced by exogenous PAF. Together with the identification of PAFR at protein level, we provide evidence that PAF is binding to a now identified PAFR in chicken macrophages. It is relevant to mention that PAFR is downregulated (and to some extent degraded) at protein level upon PAF binding in chicken primary macrophages, a well-known phenomenon in mammalian PAFR (51) that supports the present characterization of a chicken PAFR.

In contrast to what was previously observed with mouse peritoneal macrophages (56), PAF is not able to directly induce the expression of certain pro-inflammatory genes (e.g., cytokines) in chicken macrophages, although still contributing to mechanisms potentially independent of gene transcription, such as ROS release. PAF acts as an autocrine mediator of mammalian macrophage activation upon LPS challenge *via* TNF-α expression (21). Also, PAF can be biosynthesized very quickly (5, 8, 11), for example, at the very first moments of host cellular contact with bacterial LPS, which suggests that both recognition systems may operate together at the inflammatory site. Indeed, PAF is able to potentiate the pro-inflammatory response of LPS or bacteria in chicken macrophages. We could not verify whether inflammatory mediators rapidly induced by PAF (e.g., ROS) would contribute to the potentiation of LPS effects, although this hypothesis should not be discarded. Nevertheless, degradation of endogenous PAF with PAF-AH or blockade of PAFR both limit the pro-inflammatory effects of LPS alone, highlighting that LPS inflammatory response is partly dependent on PAF in chicken macrophages, as was observed in other studies in mammals (21). Therefore, paracrine- or autocrine-produced PAF is likely to play a direct role in the inflammatory response to Gram-negative bacteria-associated molecular patterns in chickens, notably at the toll-like receptor 4 (TLR4) level, responsible for the recognition of LPS. The biological relevance of these findings merits to be further investigated *in vivo*.

While some studies in mammals suggest that PAF exerts its pro-inflammatory effects through activation of p38-MAPK and NFκB (9, 21), we could not detect signs of a participation of these proteins in our experimental settings (data not shown). However, PI3K/Akt and CaM KII intracellular signaling pathways are involved in the potentiation effects of PAF on LPS stimulation, with PI3K/Akt being also essential to the pro-phagocytic effects of PAF in chicken macrophages. Both pathways have been shown to participate in different immune responses following PAFR activation in mammals (53, 56–58). CaM KII has been shown to participate in PAF reprogramming of mammalian macrophages, which would provide a stronger inflammatory response to LPS (57). Also, PAFR signaling in peritoneal macrophages requires TLR4, MyD88, and TRIF, in a so-called transactivation phenomenon (56). Although we clearly highlighted the role of CaM KII in PAFR signaling and its contribution to the LPS response, signaling pathways downstream TLR4 in chickens are not fully understood. Indeed, chickens lack a functional LPS-specific TRAM-TRIF signaling pathway, which may explain differences in response to LPS compared with mammalian species (59). Therefore, the mechanism underlying the interactions between PAFR, Cam KII, and TLR4-associated molecules in chickens remains to be fully elucidated. Of some interest, PI3K/Akt has been shown to be important to neutrophil recruitment in mammals (60). Furthermore, PAF has been shown to be important to neutrophil activation and adhesion to the endothelium (24, 52, 61). In addition to its role in macrophages, as suggested by the data in the present manuscript, the contribution of PAF to heterophil (the avian ortholog of neutrophils) arrest and recruitment *via* PI3K/Akt signaling pathway merits to be investigated.

Platelet-activating factor is directly involved in the physiopathology of cardiovascular diseases of inflammatory origin (5, 8). PAF has also been associated with EC dysfunction (53, 62). The newly established cellular model of primary chicken aortic endothelial cells (chAEC) (42) allowed us to show that PAF may directly induce increased EC monolayer permeability and strongly amplify the chAEC pro-inflammatory response to bacteria-associated molecular patterns. To our knowledge, this is the first study highlighting the role of a lipid mediator in promoting EC dysfunction in chickens. Interestingly, PAF treatment led to the upregulation of genes coding for the leukocyte adhesion molecules VCAM-1 and E-selectin, which would suggest that PAF, produced within the endothelium and adjacent tissues, could favor not only leukocyte activation but also leukocyte arrest and recruitment. Therefore, the cross-talk between PAF, macrophages, and ECs during host-response to Gram-negative bacteria could contribute to local and sustained inflammatory responses in chickens.

The physiopathology of pulmonary avian colibacillosis encompasses different inflammatory mechanisms in which exacerbated vascular leakage, heterophil and monocyte recruitment, and resident macrophage activation are believed to take place and contribute to disease severity in birds unable to control infection within a safe timeframe (33–35, 63). The contribution of recruited monocytes or tissue-resident macrophages to the host response during pulmonary colibacillosis is still being unveiled, although several studies have suggested that these resident phagocytes are involved in bacteria recognition and killing, amplification of inflammation, and, potentially, heterophil clearance (33–35, 64, 65). Here, we demonstrated that PAF is able to promote increased bacterial clearance by primary chicken macrophages *in vitro*, together with a strong amplification of the inflammatory response. Our gene expression data showed that PAFR and LPCAT2 genes are overexpressed in lungs and liver of APEC-infected chickens at early time points postinfection. While these data reveal that key components of PAF recognition and biosynthesis are locally expressed in parallel to a strong inflammatory response to APEC, we are still unable to define which cell populations are expressing these molecules. However, it is reasonable to assume that PAF is indeed produced and acts within infected sites, thereby amplifying inflammation and/or contributing to bacterial clearance as observed *in vitro*. Both PAFR and LPCAT2 genes are overexpressed in chicken macrophages and chAEC upon LPS stimulation *in vitro*, which suggests that at least these cell types may be involved in the response to colibacillosis *in vivo*.

We could not quantify PAF production in chicken tissues or cell culture supernatants due to several technical issues (i.e., contamination of closely related phospholipids during mass spectrometry analysis). However, LPCAT2 overexpression has been consistently associated with PAF biosynthesis during inflammation, while LPCAT1 activity and expression are independent of inflammatory signals (13, 66). After the activation of TLR4 by LPS, LPCAT2, but not LPCAT1, is activated and upregulated to promote PAF production in mouse macrophages (67). Indeed, LPCAT2 is believed to be the main pro-inflammatory LPCAT in the remodeling pathway for the biosynthesis of PAF in mammals. Our protein homology data suggest that chicken LPCAT2 is closely related to human and mouse counterparts. Within this scenario, we may propose that, upon contact with bacteria or bacteria-associated molecular patterns, resident macrophages, and other unidentified tissue parenchyma cells, may produce PAF. PAF would in turn amplify local inflammation through eicosanoid, cytokine, and chemokine production to facilitate initial bacterial clearance and trigger the inflammatory response. In parallel, adjacent (e.g., parenchymal) and endothelial PAF production would directly or indirectly (e.g., *via* chemokine production) promote increased leukocyte adhesion and recruitment (e.g., heterophils and monocytes), together with increased vascular leakage, therefore, contributing to the orchestration of classical cellular and molecular events that take place at the onset of the inflammatory response. We therefore suggest that PAF might be an important mediator of inflammation in the host response to Gram-negative bacteria causing economically relevant diseases in poultry farms worldwide, such as APEC.

A PAF receptor antagonist, Modipafant, was already used in the past to treat asthma, although its use is currently discontinued (68). There is also an ongoing clinical trial to validate the use of Modipafant as a therapeutic strategy to limit exacerbated inflammation observed in viral hemorrhagic diseases such as Dengue fever.^5^ Preliminary experiments suggest that PAFR antagonists would be active *in vivo* in chickens (data not shown), and a proof of concept on the use of PAFR antagonists to limit exacerbated inflammation associated with experimental colibacillosis are currently being performed. Although the feasibility of such therapeutic strategy in the poultry industry is still difficult to envisage, these experiments will bring biologically relevant information on how lipid mediators and their receptors could be pharmacologically manipulated in chickens, so as to pave the way to alternative therapies in face of the widespread antibiotic resistance in poultry farms and the lack of efficacious vaccines for certain avian diseases.

In conclusion, we believe that the present study sheds light on the role and function of PAF in bridging endothelium function and macrophage-associated innate immunity in chickens in response to bacteria-derived stimuli, a hitherto unknown mechanism in birds. Also, these activity profiles may be shared by other yet to be identified lipid mediators of inflammation, a line of research that should be of interest to the scientific community in the fields of avian immunology, evolution, and host–pathogen interaction.

## Ethics Statement

All animals used in the avian pathogenic *Escherichia coli* (APEC) infection protocol were treated according to EU recommendations for animal welfare, and the protocol was approved by the French regional ethics committee number 19 (Comité d’Ethique en Expérimentation Animale Val de Loire) under the reference CL2007-44.

## Author Contributions

RG designed and performed the experiments, analyzed data, and wrote the manuscript. DG, NC, AT, and AL performed the experiments and analyzed data. GB and EE performed the experiments. ST, PQ, and CS contributed reagents, materials, intellectual input, and revised the manuscript.

## Conflict of Interest Statement

The authors declare that the research was conducted in the absence of any commercial or financial relationships that could be construed as a potential conflict of interest. The reviewer TK and handling editor declared their shared affiliation.

## Footnotes

^1^http://www.ncbi.nlm.nih.gov/tools/primer-blast.

^2^http://www.uniprot.org/blast/.

^3^http://www.cbs.dtu.dk/services/NetPhos/.

^4^http://www.cbs.dtu.dk/services/NetNGlyc/.

^5^https://clinicaltrials.gov/ct2/show/NCT02569827.
