# Supplementary figures and images for: Characterization of the Phospholipid Platelet-Activating Factor As a Mediator of Inflammation in Chickens
Source: Front Vet Sci. 2017 Dec 18;4:226. doi: 10.3389/fvets.2017.00226 (PMC5741692; doi:10.3389/fvets.2017.00226)

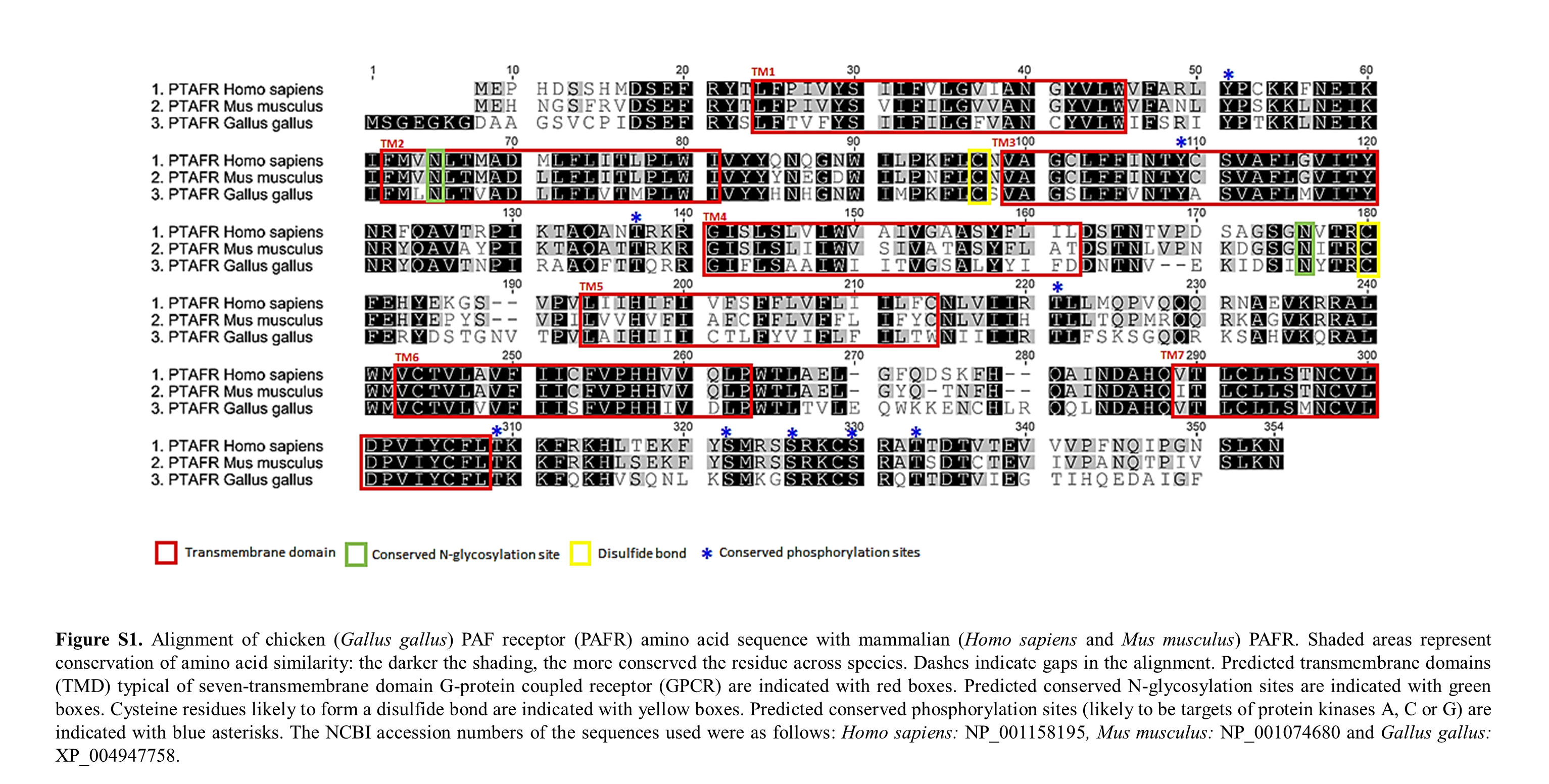

Supplement: Supplementary file 1 [file Image_1.tif]

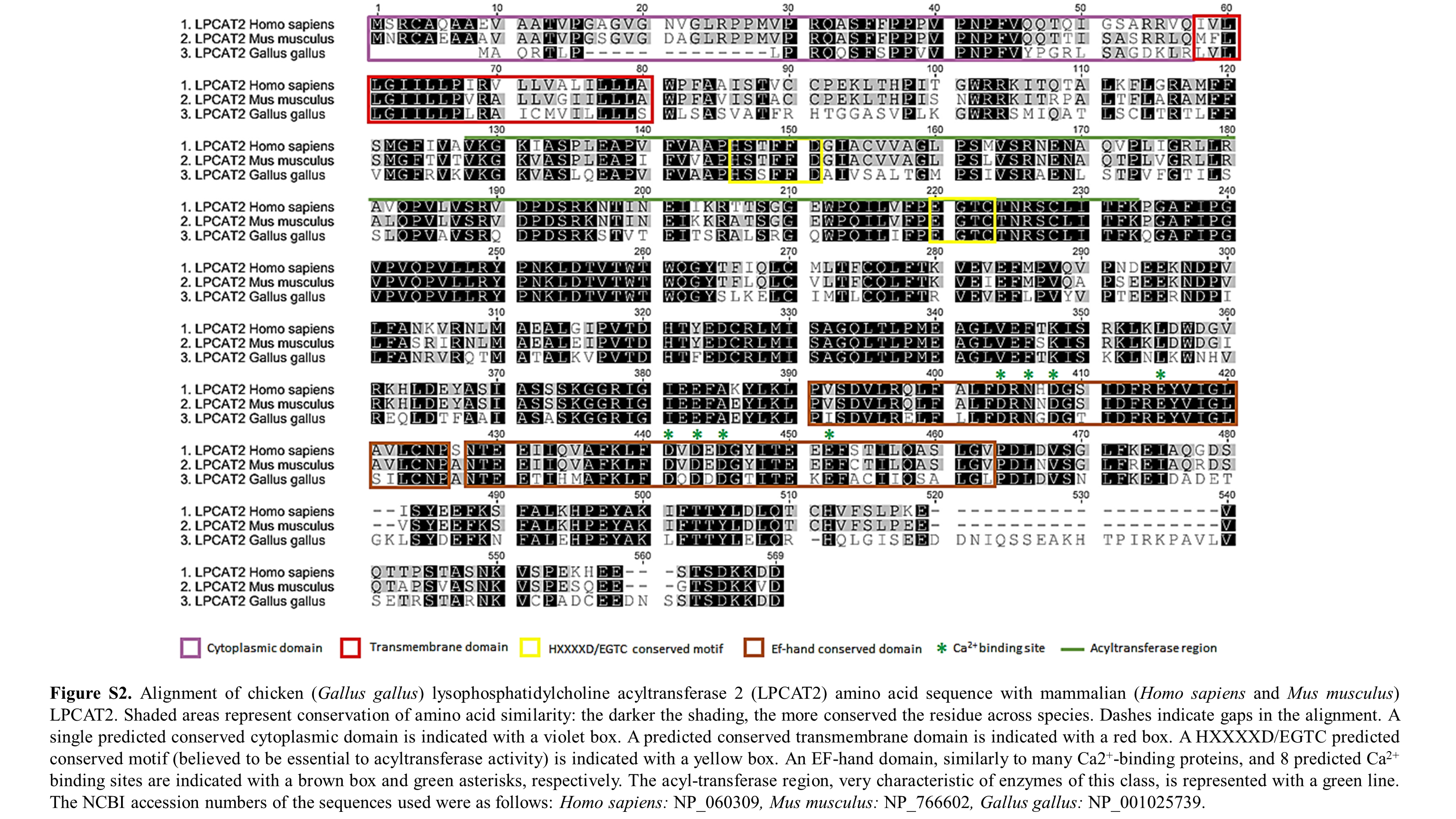

Supplement: Supplementary file 2 [file Image_2.tif]
